# Conserved role of Atx2 in JNK pathway activation

**DOI:** 10.1038/s41419-026-08797-9

**Published:** 2026-05-02

**Authors:** Xinyao Li, Xiaojie Zhu, Wenzhe Li, Hansong Deng, Lei Xue

**Affiliations:** 1 Department of Nuclear Medicine, Shanghai Tenth People’s Hospital, Shanghai Key Laboratory of Signaling and Diseases Research, School of Life Sciences and Technology, Tongji University, Shanghai, China; 2 Yangzhi Rehabilitation Hospital, Sunshine Rehabilitation Center, Frontier Science Center for Stem Cell Research, School of Life Sciences and Technology, Tongji University, Shanghai, China

**Keywords:** Drosophila, Apoptosis

## Abstract

The c-Jun N-terminal kinase (JNK) pathway is an evolutionarily conserved signaling cascade that regulates development, stress responses, and pathogenesis. While aberrant JNK activation is linked to cancer and neurodegeneration, its regulatory mechanisms are not fully understood. Here, we identify the RNA-binding protein Ataxin-2 (Atx2) as a novel, essential regulator of JNK-mediated cell death and migration in *Drosophila*. Atx2 deficiency suppressed JNK-dependent apoptosis, tumor growth and invasion, and thorax closure in normal development, while its overexpression activated JNK signaling, promoting cell death, migration, and tissue remodeling. Mechanistically, Atx2 binds the 3′ UTR of *hipk* mRNA, stabilizing it to enhance the expression of Hipk, a core upstream JNK kinase. Strikingly, this mechanism is conserved: human ATXN2L potently activated Hipk-JNK signaling and cell death in *Drosophila* and HeLa cells. Our findings reveal a conserved post-transcriptional mechanism for JNK pathway regulation and nominate Atx2 family proteins as potential therapeutic targets in JNK-associated pathologies.

## Introduction

Ataxin-2 (Atx2) is a conserved cytoplasmic RNA-binding protein (RBP) with essential roles in post-transcriptional regulation. It participates in diverse processes, including RNA metabolism, calcium signaling, circadian rhythm regulation, and stress granule (SG) assembly [1–3]. Structurally, Atx2 contains two N-terminal domains, Sm-like (Lsm) and Lsm-associated (LsmAD), together with a C-terminal poly(A)-binding protein 1 (PABP1)-interacting motif 2 (PAM2), flanked by intrinsically disordered regions (IDRs) [4]. The Lsm and LsmAD domains mediate RNA binding and regulate transcript stability and translation, while IDRs promote ribonucleoprotein (RNP) and SG assembly [5, 6]. Through its PAM2 motif, Atx2 interacts with PABP to modulate translation [7]. Although the yeast homolog Pbp1 lacks a PAM2 motif, it retains a functional interaction with Pab1 (the yeast PABPC1 ortholog), underscoring the evolutionary conservation of Atx2 functions [8, 9].

Due to its Lsm domain architecture, Atx2 is classified within the Lsm protein family, which broadly regulates RNA splicing, degradation, and translation [10]. In humans, expansion of the polyglutamine (polyQ) tract in ATXN2 (normally 22–23 repeats, expanded to ≥34) causes spinocerebellar ataxia type 2 (SCA2) and has been associated with amyotrophic lateral sclerosis (ALS), Huntington’s disease (HD), and Parkinson’s disease (PD) [11–14]. While ATXN2’s roles in neurons and neurodegeneration are well established, its physiological and pathological functions outside the nervous system remain less defined.

The c-Jun N-terminal kinase (JNK) pathway is a conserved branch of the mitogen-activated protein kinase (MAPK) cascade that governs apoptosis, proliferation, tissue remodeling, aging, and tumorigenesis [15]. In *Drosophila*, JNK activation is triggered by developmental and stress cues through a core module comprising the TNF-family ligand Eiger (Egr), its receptors Wengen and Grindelwald, MAP3Ks such as dTak1 and Wallenda (Wnd), the MAP2K Hemipterous (Hep), and the JNK ortholog Basket (Bsk) [16–18]. Once activated, JNK phosphorylates downstream effectors to elicit context-dependent responses, including apoptosis and compensatory proliferation [19]. A well-characterized model of JNK-induced apoptosis is Egr overexpression in the developing eye, which drives strong JNK activation and results in a small-eye phenotype due to excessive cell death [20, 21].

Although polyQ-expanded ATXN2 has been reported to enhance caspase activation [22], its role in regulating JNK signaling has not been defined. Here, we identify Atx2 as a novel, positive regulator of canonical JNK signaling during *Drosophila* development. Genetic and molecular analyses show that *Atx2* depletion suppresses JNK-mediated apoptosis, tumor growth, and invasion, whereas Atx2 overexpression activates JNK signaling and promotes JNK-dependent cell death and migration. Mechanistically, Atx2 enhances JNK activity by binding and stabilizing *hipk* mRNA, which encodes a critical upstream kinase in the pathway. Importantly, this regulatory function of Atx2 is evolutionarily conserved in the human ortholog ATXN2L, but not a poly(Q)-expanded ATXN2. Together, these findings identify Atx2 family proteins as novel, conserved regulators of JNK signaling, with critical roles in development and tumorigenesis.

## Results

### Atx2 promotes Egr-induced cell death in *Drosophila* eyes

**Fig. 1:**
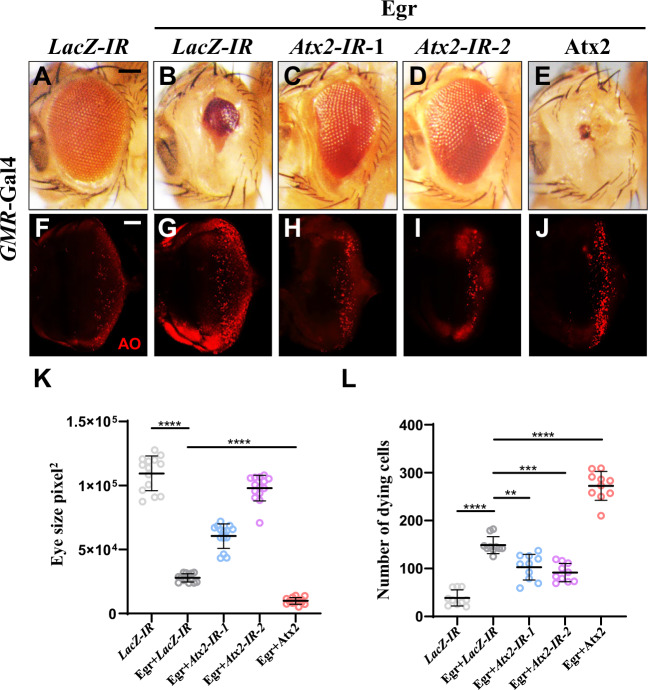
Atx2 promotes Egr-induced cell death in *Drosophila* eyes. **A**–**E** Light micrographs of adult eyes. Compared with the control (**A**), *GMR* > Egr induced a small-eye phenotype (**B**), which was suppressed by two independent *Atx2 RNAi* lines (**C**, **D**) and enhanced by Atx2 overexpression (**E**). **F**–**J** Fluorescence micrographs of acridine orange (AO) staining in third instar larval eye discs. Compared with control (**F**), ectopic Egr expression increased cell death (**G**), which was suppressed by *Atx2* knockdown (**H**, **I**) and enhanced by Atx2 overexpression (**J**). Quantification of adult eye size (*n* = 14 per group) (**K**) and AO-positive cell numbers (*n* = 10 per group) (**L**). Data are mean ± SEM. One-way ANOVA: ****p < 0.0001, ***p < 0.001, **p < 0.01. Scale bars: 100 μm (**A**–**E**), 50 μm (**F**–**J**).

Ectopic expression of Egr under *GMR*-Gal4 activates JNK-dependent cell death, producing a small-eye phenotype in *Drosophila* adults [23]. In a genetic screen for modifiers of this phenotype, we found that *RNAi*-mediated knockdown of *Atx2* markedly suppressed the *GMR* > Egr small-eye phenotype. Consistent with previous reports, Egr overexpression induced extensive cell death in third-instar eye discs (visualized by Acridine Orange staining) and reduced adult eye size (Fig. 1B, G) relative to controls (Fig. 1A, F) [24]. While overexpression or depletion of *Atx2* alone driven by *GMR*-Gal4 had no effect on eye size (Fig. S1), knockdown of *Atx2* using two independent *RNAi* lines suppressed both the Egr-induced cell death (Fig. 1H, I) and the small-eye phenotype (Fig. 1C, D). Conversely, overexpression of Atx2 further exacerbated these phenotypes (Fig. 1E, J). Quantitative analyses are present in Fig. 1K, L, and the efficacy of *Atx2* knockdown was validated in Fig. S7A.

Egr activates JNK via the dTak1-Hep-Bsk kinase cascade [23]. Overexpression of dTak1 driven by *sev*-Gal4 induced a small, rough-eye phenotype that was suppressed by *Atx2* knockdown (Fig. S2A–C), whereas *GMR*>Hep^CA^-induced small eyes were unaffected (Fig. S2D–F), placing Atx2 downstream of dTak1 but upstream of, or parallel to, Hep in promoting JNK signaling.

### Atx2 activates JNK signaling and induces JNK-dependent cell death

To test whether Atx2 is sufficient to activate JNK, we used the *patched* (*ptc*)-Gal4 driver to express Atx2 along the anterior/posterior (A/P) boundary of wing imaginal discs. This resulted in strong upregulation of the JNK pathway reporter *puc*-LacZ (Fig. S3A, C) [25]. In addition, Atx2 overexpression robustly activated the expression of *TRE*-RFP (Fig. S3B-B”, D-D”), a reporter for AP-1 transcriptional activity [26, 27]. Notably, *TRE*-RFP expression extended beyond GFP-positive Atx2-expressing cells into neighboring GFP-negative cells (Fig. S3D-D”), indicating both cell-autonomous and non-autonomous activation of JNK signaling. This non-autonomous propagation of JNK activity is a well-documented phenomenon in *Drosophila* development [28–30].

To directly and quantitatively assess JNK activation, we performed western blot analysis to measure JNK phosphorylation in third instar larvae. Overexpression of Atx2 significantly increased levels of phosphorylated JNK (p-JNK) without affecting total JNK levels (Fig. S3E, F), confirming that Atx2 is sufficient to promote JNK activation in vivo.

**Fig. 2:**
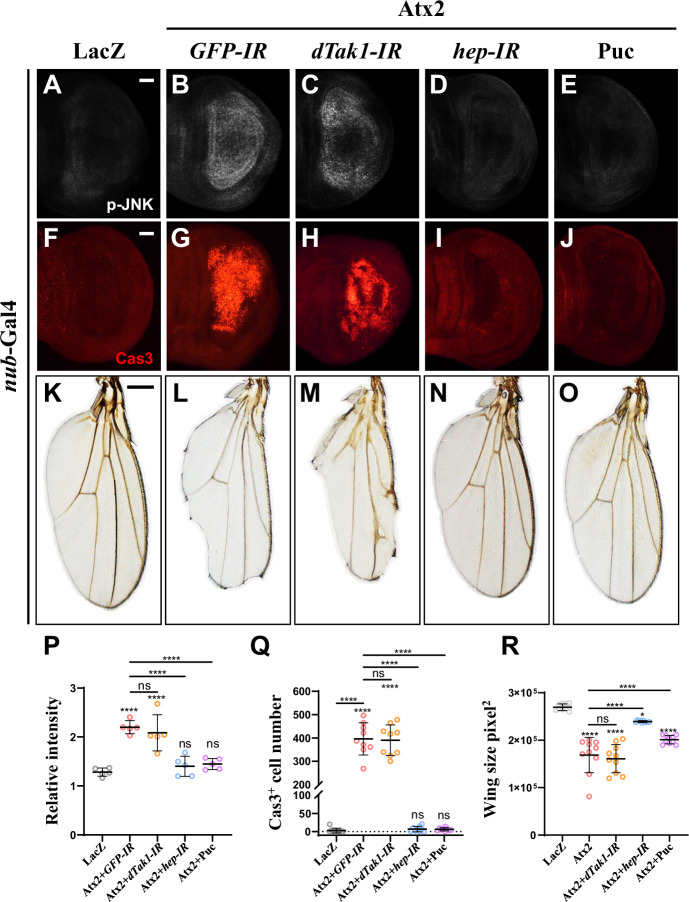
Atx2 induces JNK-dependent cell death. **A**–**E** Fluorescence micrographs of wing discs stained for phosphorylated JNK (p-JNK). Compared with control (**A**), Atx2 overexpression induced p-JNK accumulation (**B**). This induction was unaffected by *dTak1 RNAi* (**C**) but suppressed by *hep RNAi* (**D**) or Puc overexpression (**E**). **F**–**J** Fluorescence images of cleaved Caspase-3 staining. Compared with control (**F**), Atx2 overexpression induced Caspase-3 activation (**G**), which was unaffected by *dTak1 RNAi* (**H**) but suppressed by *hep RNAi* (**I**) or Puc overexpression (**J**). **K**–**O** Light micrographs of adult wings. Compared with control (**K**), *nub* > Atx2 caused a small, notched wing phenotype (**L**), which was not rescued by *dTak1 RNAi* (**M**) but was suppressed by *hep RNAi* (**N**) and Puc overexpression (**O**). Quantification of p-JNK intensity (*n* = 5 per group) (**P**), number of Caspase-3-positive cells (*n* = 9 per group) (**Q**), and adult wing size (*n* = 10 per group) (**R**). Data are mean ± SEM. One-way ANOVA or Mann–Whitney test: ****p < 0.0001, ***p < 0.001; n.s., not significant. Scale bars: 50 μm (**A**–**J**), 250 μm (**K**–**O**).

To determine whether Atx2-induced JNK activation leads to apoptosis, we overexpressed Atx2 in the wing pouch using the *nubbin* (*nub*)-Gal4 driver. This resulted in elevated p-JNK levels (Fig. 2A, B, P), widespread apoptosis as indicated by cleaved Caspase-3 staining (Fig. 2F, G, Q), and a notched, reduced wing phenotype in adult flies (Fig. 2K, L, R). These phenotypes were suppressed by co-expression of Puc, a negative regulator of JNK signaling (Fig. 2E, J, O–R), indicating that they are JNK-dependent. Atx2-induced phenotypes were not affected by knockdown of *dTak1* (Fig. 2C, H, M, P–R) or *wnd* (Fig. S4A–C, E–G, I–K, M–O), but were significantly suppressed by depletion of *hep* (Fig. 2D, I, N, P–R) or *bsk* (Fig. S4D, H, L, M–O). Together, these findings support the conclusion that Atx2 acts downstream of dTak1 or Wnd and upstream of Hep to promote JNK-mediated cell death.

### Loss of *Atx2* suppresses JNK-driven tumor growth and invasion

**Fig. 3:**
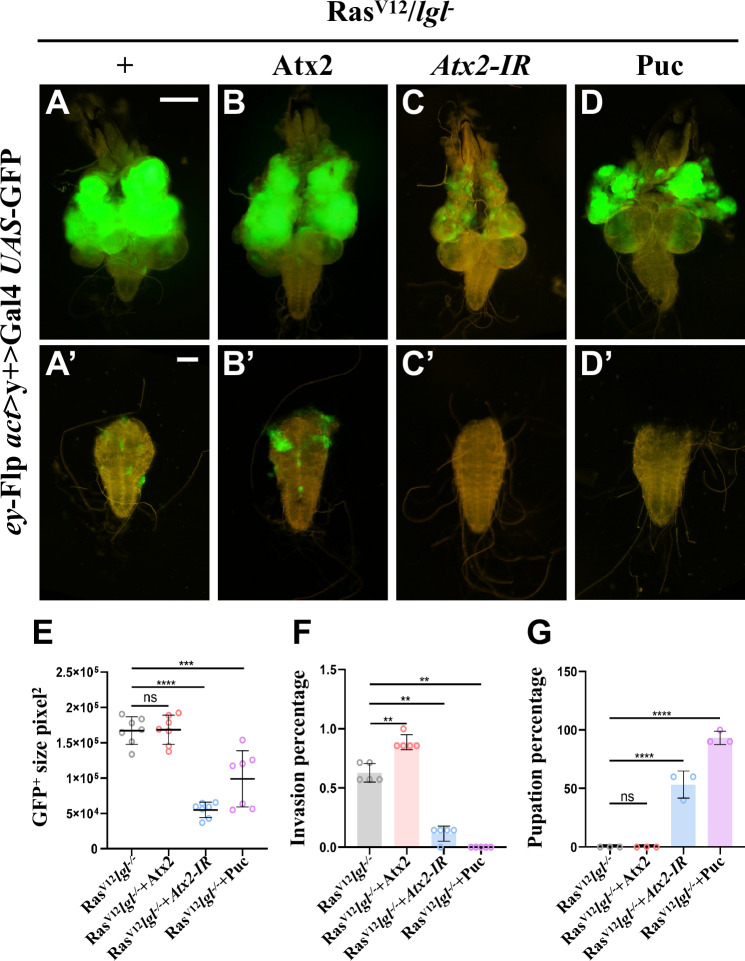
Atx2 regulates tumor growth and invasion. **A**–**D** Fluorescence images of GFP-marked Ras^V12^/*lgl*^*-*^ MARCM clones in cephalic complexes. Clones exhibited tumor overgrowth (**A**). Atx2 overexpression did not affect tumor size (**B**) but increased invasion into the ventral nerve cord (VNC, **B′**). *Atx2* knockdown suppressed both tumor size (**C**) and VNC invasion (**C′**), and improved pupation rate (**G**). Puc overexpression similarly reduced tumor size (**D**) and invasion (**D′**), and improved pupation (**G**). Quantification of GFP-positive tumor area (*n* = 7 per group) (**E**), VNC invasion frequency (*n* = 35 per group, each point represents 7 samples) (**F**), and pupation rate (*n* = 30 per group, each point represents 10 samples) (**G**). Data are mean ± SEM. One-way ANOVA and Mann-Whitney test: ****p < 0.0001, *p < 0.05, n.s., not significant. Scale bars: 250 μm (**A**–**D**), 100 μm (**A′**–**D′**).

In a Ras^V12^/*lgl*^−^ model of JNK-dependent tumorigenesis [31, 32], eye disc clones formed large, invasive tumors that invaded the ventral nerve cord (VNC) (Fig. 3A, A′, E, F) and caused larval lethality (Fig. 3G). Puc overexpression suppressed tumor growth, invasion, and lethality (Fig. 3D, D′, E–G), confirming that they are JNK-dependent. Strikingly, *Atx2* knockdown similarly reduced tumor size, blocked VNC invasion, and increased pupation rates (Fig. 3C, C′, E–G). Although co-expression of Atx2 did not further increase tumor size (Fig. 3B, E), it markedly enhanced invasion frequency (Fig. 3B′, F). Collectively, these findings indicate that Atx2 is required for JNK-dependent tumor growth and invasion.

### Atx2 regulates JNK signaling through *hipk*

To elucidate how Atx2 regulates JNK activity, we analyzed published data and identified the kinase Hipk as a strong candidate target of Atx2. A recent TRIBE (Targets of RNA-Binding Proteins Identified by Editing) study profiling Atx2-bound mRNAs in *Drosophila* adult brains and S2 cells identified *hipk* mRNA among 12 overlapping targets [33]. Given that Hipk is a known positive regulator of Hep-JNK signaling [34], we hypothesize that Atx2 promotes JNK signaling by stabilizing *hipk* mRNA.

**Fig. 4:**
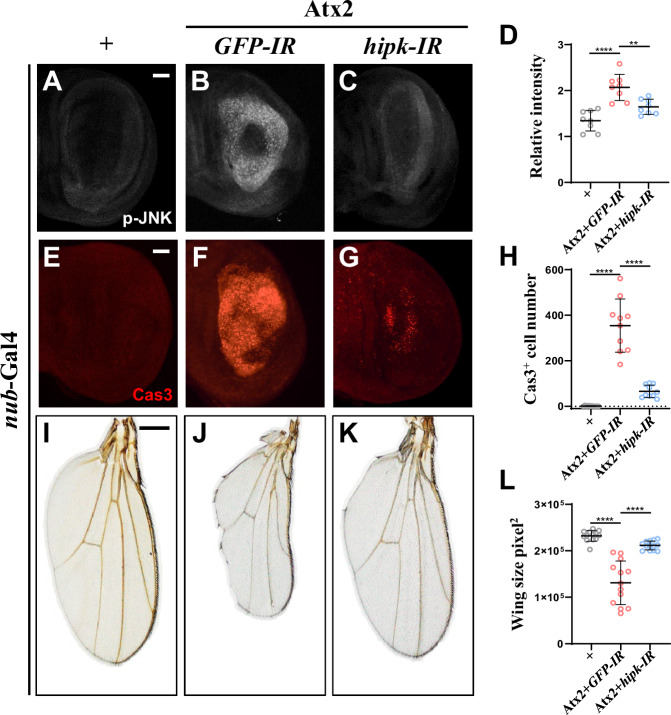
Atx2 activates JNK signaling via Hipk. **A**–**C** Fluorescence micrographs of wing discs stained for p-JNK. Compared with control (**A**), Atx2 overexpression increased p-JNK levels (**B**), which was suppressed by *hipk RNAi* (**C**). **E**–**G** Fluorescence micrographs of cleaved Caspase-3 staining. Compared with control (**E**), Atx2 overexpression increased apoptosis (**F**), which was suppressed by *hipk RNAi* (**G**). **I**–**K** Light micrographs of adult wings. Compared with control (**I**), Atx2 overexpression caused a small, notched wing phenotype (**J**), which was rescued by *hipk* knockdown (**K**). Quantification of p-JNK intensity (*n* = 8 per group) (**D**), Caspase-3-positive cells (*n* = 10 per group) (**H**), and adult wing size (*n* = 13 per group) (**L**). Data are mean ± SEM. One-way ANOVA and Mann-Whitney test: ****p < 0.0001, **p < 0.01. Scale bars: 50 μm (**A**–**C**, **E**–**G**), 250 μm (**I**–**K**).

**Fig. 5:**
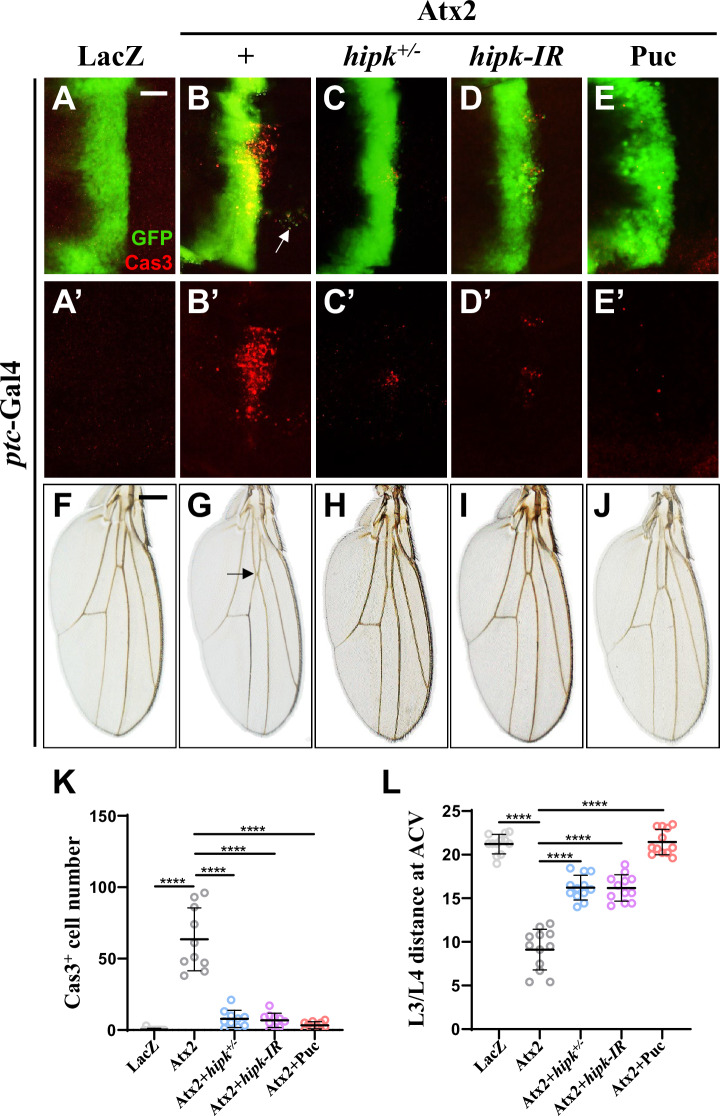
Atx2 promotes *Hipk*-dependent cell death and invasion. (**A**–**E**, **A′**–**E′**) Fluorescence images of wing discs. The *ptc* expression domain is marked by GFP (**A**–**E**). Cleaved Caspase-3 staining (**A′**–**E′**) shows that Atx2 overexpression induced cell migration (arrow in **B**) and apoptosis (**B′**). These effects were suppressed by a heterozygous *hipk* mutation (**C**, **C′**), *hipk RNAi* (**D**, **D′**), or Puc overexpression (**E**, **E′**). **F**–**J** Adult wings showing that *ptc* > Atx2 caused tissue loss between L3 and L4 veins (**G**, arrow indicates reduced anterior cross vein, ACV), which was rescued by *hipk* depletion (**H**, **I**) or Puc overexpression (**J**). Quantification of Caspase-3-positive cells (*n* = 10 per group) (**K**) and L3-L4 vein spacing at the ACV (*n* = 12 per group) (**L**). Data are mean ± SEM. One-way ANOVA or Mann-Whitney test: ****p < 0.0001. Scale bars: 25 μm (**A**–**E**, **A′**–**E′**), 250 μm (**F**–**J**).

We first confirmed that ectopic Hipk expression driven by *ptc-*Gal4 activated JNK signaling and reduced the L3-L4 wing area (Fig. S5). *hipk* knockdown strongly suppressed *nub* > Atx2-induced p-JNK elevation (Fig. 4A–D), apoptosis (Fig. 4E–H), and wing-size reduction (Fig. 4I–L). Consistent with earlier reports that JNK activation at the A/P boundary of wing discs drives apoptosis and invasive migration [35], *ptc* > Atx2 induced robust cell migration (Fig. 5B) and cleaved caspase-3 (CC-3) signal (Fig. 5B′) in larval wing discs, as well as tissue loss between L3 and L4 veins in adult wings (Fig. 5G). These phenotypes were rescued by heterozygous *hipk* mutation, *hipk* knockdown, or co-expression of Puc (Fig. 5C–E, C′–E′, H–L). Together, these results demonstrate that Hipk is essential for Atx2-mediated JNK activation, apoptosis, and cell migration.

Finally, we confirmed that apoptosis is the primary driver of the wing phenotype. Co-expression of the anti-apoptotic protein P35 significantly rescued both *ptc* *>* *Atx2*-induced apoptosis in larval wing discs and tissue loss in adult wings (Fig. S6), indicating that the Atx2 overexpression phenotype is a direct consequence of programmed cell death.

### Atx2 stabilizes *hipk* mRNA via its 3′UTR

**Fig. 6:**
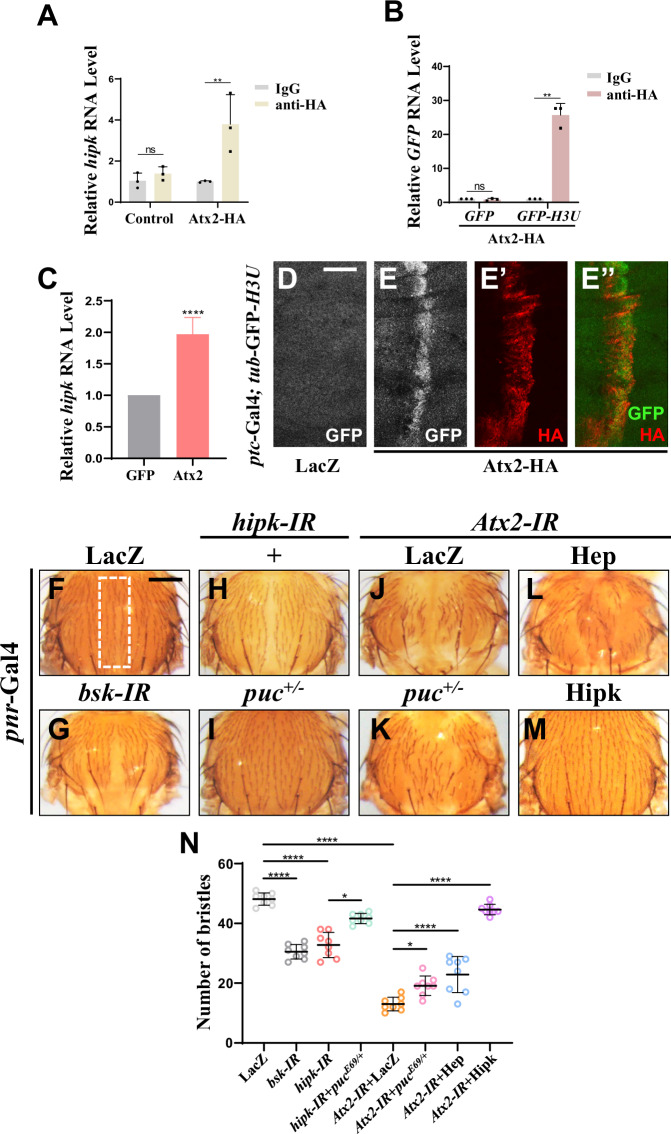
Atx2 stabilizes *hipk* mRNA and promotes physiological JNK-dependent thorax closure. **A** RNA immunoprecipitation (RIP) assay showing enrichment of *hipk* mRNA in Atx2-HA immunoprecipitates compared with IgG control. The control plasmid was pUAST-3×HA, an empty vector (*n* = 3 per group). **B** RIP of S2 cells co-expressing HA-Atx2 with GFP or GFP fused to the *hipk* 3′ UTR (GFP-*H3U*), showing preferential binding of Atx2 to the *hipk* 3′ UTR (*n* = 3 per group). **C** RT-qPCR analysis shows Atx2 overexpression in larvae significantly increased endogenous *hipk* mRNA levels (*n* = 3 per group). Atx2 driven by *hs*-GAL4, 1/3 L3 larva tissues were lysed in NucleoZOL 6H after heatshock to extract RNA. **D**, **E** Wing discs expressing the *tub*-GFP-*H3U* reporter (*n* = 5 per group). Compared with control (**D**), Atx2 overexpression driven by *ptc*-Gal4 markedly increased GFP reporter intensity (**E**–**E”**). HA staining indicates the Atx2 expression pattern (**E′**); merged images are shown in (**E″**). **F**–**M** Light micrographs of adult thoraces. Compared with control (**F**), *bsk* depletion caused thorax closure defects (**G**). Similar defects were observed with knockdown of *hipk* (**H**) or *Atx2* (**J**), which were rescued by a heterozygous *puc* mutation (**I**, **K**) and co-expression of Hep (**L**) or Hipk (**M**). **N** Quantification of thoracic bristle numbers within the boxed region in (**F**) (*n* = 8 per group). Data are mean ± SEM. One-way ANOVA: ****p < 0.0001, *p < 0.05. Scale bar: 50 μm (**D**–**E**), 200 μm (**F**–**M**).

Atx2 is known to bind 3′UTRs of target mRNAs to enhance transcript stability [5, 35]. RNA immunoprecipitation (RIP) assays in S2 cells demonstrated that HA-tagged Atx2 specifically associated with *hipk* mRNA (Fig. 6A). Consistently, Atx2-HA preferentially bound a *GFP* reporter mRNA containing the *hipk* 3′UTR (*GFP*-*H3U*), but not a *GFP* transcript lacking this 3’UTR (*GFP*) (Fig. 6B), indicating that Atx2 directly interacts with the *hipk* 3′UTR.

To validate this interaction in vivo, we examined *hipk* transcript levels in third instar larvae and found that Atx2 overexpression significantly increased *hipk* mRNA abundance (Fig. 6C). Consistently, in transgenic flies expressing a GFP reporter fused to the *hipk* 3′UTR under the control of the tubulin promoter (*tub*-GFP-*H3U*), Atx2 overexpression driven by *ptc*-Gal4 markedly enhanced GFP fluorescence along the anterior-posterior boundary in wing discs (Fig. 6D, E). Conversely, depletion of *Atx2* reduced *hipk* mRNA level (Fig. S7A) and the GFP fluorescence within the *ptc*-Gal4 expression domain (Fig. S7B–D).

To exclude the possibility that Atx2 directly regulates *GFP* mRNA stability or translation, we expressed Atx2 in an *act* > GFP background. Compared with control, overexpression of Atx2 did not affect GFP expression at either the mRNA (Fig. S7E) or protein levels (Fig. S7F). Together, these results demonstrate that Atx2 stabilizes *hipk* mRNA through direct interaction with its 3′UTR.

### Atx2 regulates physiological JNK signaling in normal development

Given the requirement of Atx2 for ectopic JNK-induced apoptosis, migration and tumorigenesis, we next examined whether it also contributes to physiological JNK activity during normal development. Thorax closure in *Drosophila*, an extensively characterized morphogenetic event, involves cell movements that are strictly dependent on JNK signaling [36, 37]. As expected, knockdown of *bsk* with *pnr*-Gal4 resulted in a thorax cleft phenotype, characterized by a loss of midline bristles (Fig. 6G), compared with the control (Fig. 6F). Strikingly, depletion of either *hipk* or *Atx2* produced similar thorax closure defects (Fig. 6H, J), which were rescued by enhanced JNK activity in heterozygous *puc* mutants (Fig. 6I, K). Moreover, in line with the genetic epistasis analyses described above, Atx2 knockdown-induced thorax clefts were significantly suppressed by co-expression of Hep or Hipk (Fig. 6L, M). Together, these results demonstrate that Atx2 is required for promoting physiological Hipk-Hep-JNK signaling during thorax closure, thereby linking Atx2 to normal developmental morphogenesis. This physiological role, combined with its functions in JNK-mediated apoptosis, migration and tumorigenesis, highlights Atx2 as a central regulator of JNK pathway activity across diverse biological contexts.

### Evolutionary conservation of JNK regulation by human Atx2 ortholog ATXN2L

**Fig. 7:**
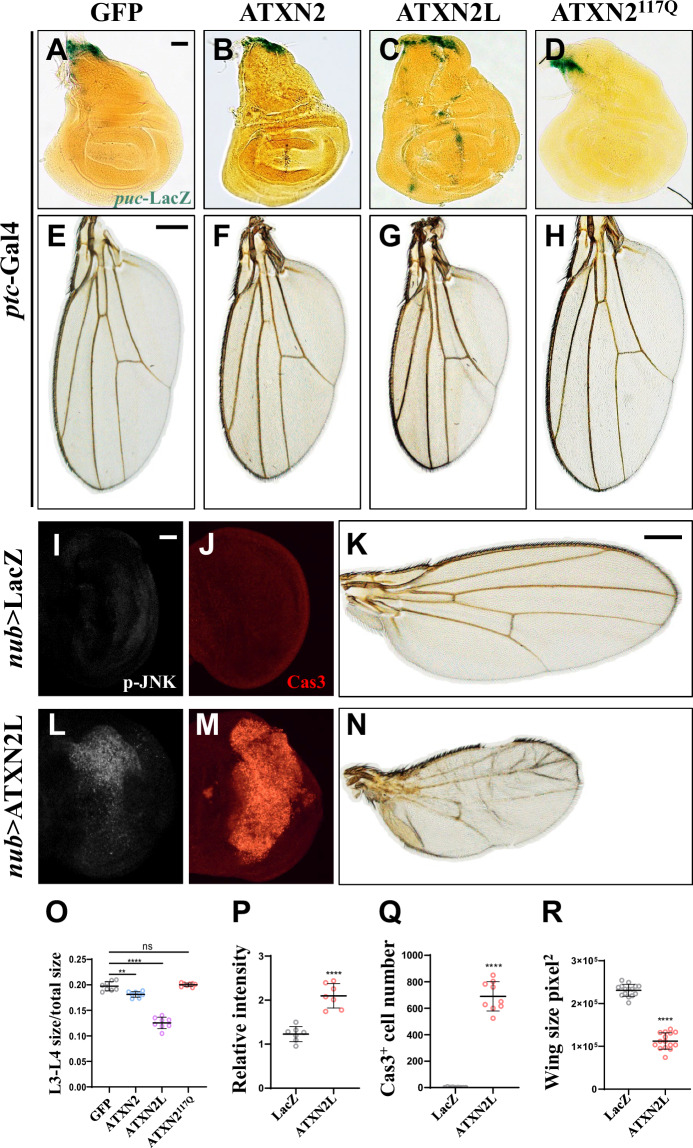
Human ATXN2 and ATXN2L activate JNK signaling and induce cell death in *Drosophila.* Light micrographs of wing discs (**A**–**D**) and adult wings (**E**–**H**). Compared with control (**A**, **E**), *ptc*-Gal4 driven overexpression of human ATXN2 or ATXN2L, but not the expanded ATXN2^117Q^, activated the JNK reporter *puc*-LacZ along the A/P boundary in larval wing discs (*n* = 5 per group) (**B**–**D**) and reduced L3–L4 area in adult wings (**F**–**H**). Fluorescence images of wing discs (**I**, **J**, **L**, **M**) and light micrographs of adult wings (**K**, **N**). Compared with controls (**I**–**K**), overexpression of ATXN2L by *nub*-Gal4 induced p-JNK accumulation (**L**), Caspase-3 activation (**M**), and a small wing phenotype (**N**). Quantification of the L3-L4 area relative to total wing area (*n* = 8 per group) (**O**), p-JNK intensity (*n* = 7 per group) (**P**), Caspase-3-positive cells (*n* = 9 per group) (**Q**), and adult wing size (*n* = 15 per group) (**R**). Data are mean ± SEM. One-way ANOVA or Unpaired *t*-test or Mann-Whitney test: ****p < 0.0001, ***p < 0.001, **p < 0.01, n.s., not significant. Scale bars: 50 μm (**A**–**D**, **I**–**J**, **L**–**M**), 250 μm (**E**–**H**, **K**, **N**).

To investigate the evolutionary conservation of Atx2-mediated JNK regulation, we expressed the human Atx2 orthologs ATXN2 and ATXN2L in *Drosophila*. Expression of either protein, but not a polyQ-expanded form (ATXN2^117Q^), driven by *ptc*-Gal4 activated JNK signaling along the A/P boundary in larval wing discs (Fig. 7A–D) and reduced the L3-L4 intervein area in adult wings (Fig. 7E–H, O). These results indicate that the JNK-regulatory function of Atx2 is conserved in its human orthologs but is disrupted by polyQ expansion.

**Fig. 8:**
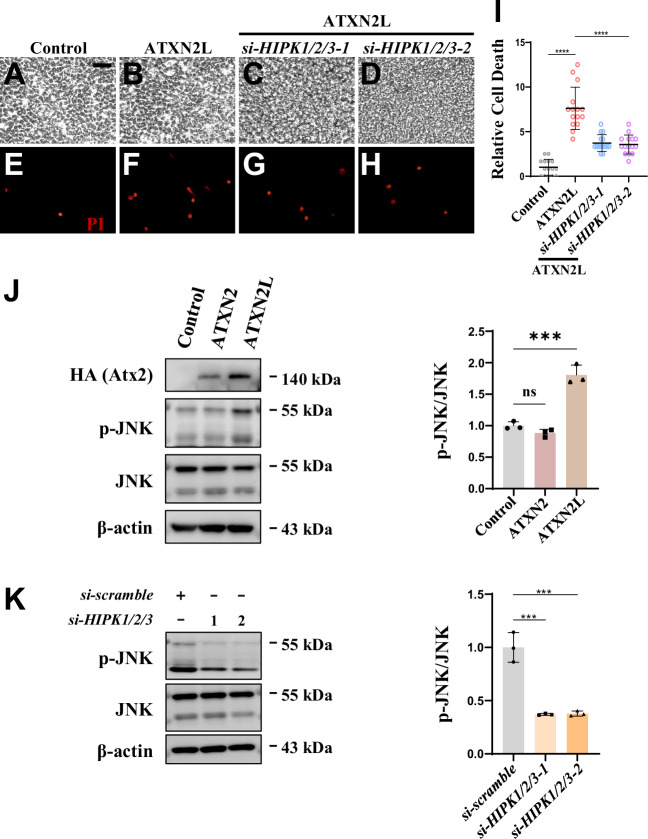
ATXN2L induces *HIPK*-dependent JNK activation and cell death in HeLa cells. Light micrographs (**A**–**D**) and fluorescence images (**E**–**H**) of HeLa cells. Compared with control (**A**, **E**), ATXN2L overexpression increased propidium iodide (PI)-positive cell death (**B**, **F**), which was suppressed by two independent *HIPK1/2/3 RNAi* combinations (**C**, **D**, **G**, **H**). **I** Quantification of PI-positive HeLa cells (*n* = 15 per group). **J** Immunoblot analysis shows that ATXN2L, but not ATXN2, upregulated the relative JNK phosphorylation level, as indicated by the p-JNK/JNK ratio. HeLa cells overexpressing ATXN2 or ATXN2L were lysed in RIPA buffer for protein extraction. (*n* = 3 per group) **K** Simultaneous depletion of *HIPK1/2/3* reduced p-JNK levels. (*n* = 3 per group) Data are mean ± SEM. One-way ANOVA: ****p < 0.0001, ***p < 0.001; n.s., not significant. Scale bars: 50 μm (**A**–**H**).

Based on its higher sequence identity to Atx2 and stronger ability to activate JNK signaling, ATXN2L was selected for further analysis. Ectopic expression of ATXN2L driven by *nub*-Gal4 robustly phenocopied the effects of Atx2, leading to JNK phosphorylation, apoptosis, and a notched small-wing phenotype (Fig. 7I-N, P–R). This conserved function was further veridated in HeLa cells, where ATXN2L overexpression triggered JNK phosphorylation (Fig. 8J) and cell death (Fig. 8A, B, E, F). Notebly, both phenotypes were suppressed by co-depletion of *HIPK1*, *HIPK2*, and *HIPK3* (Fig. 8C, D, G–I), establishing their dependence on HIPK. Efficient knockdown of *HIPKs* was validated (Fig. S8), and deletion of *HIPKs* reduced JNK phosphorylation levels (Fig. 8K). Consistent with our findings in *Drosophila*, depletion of *ATXN2L* reduced the mRNA level of *HIPKs* (Fig. S9B) and attenuated JNK phosphorylation in HeLa cells (Fig. S9C). In contrast, modulation of ATXN2 expression did not affect JNK activation (Fig. 8J, Fig. S9C) or *HIPK* mRNA levels in HeLa cells (Fig. S9A), suggesting that ATXN2L, but not ATXN2, retains the ability to activate JNK signaling through stabilization of *HIPK* mRNA in this cellular context.

To further assess the functional relevance of ATXN2L, we analyzed CancerSEA datasets and found that ATXN2L, but not ATXN2, was positively correlated with epithelial-mesenchymal transition (EMT) and metastasis signatures in acute myeloid leukemia (AML) (Fig. S10A) [38]. Consistently, Transwell assays revealed that ATXN2L overexpression promoted cell migration (Fig. S10B, C) and invasion (Fig. S10G, H) in HeLa cells. Importantly, ATXN2L-induced migration and invasion were suppressed by co-depletion of *HIPK1/2/3* (Fig. S10E, F, J, K), indicating that these effects are HIPK dependent.

In conclusion, the core function of Atx2 in promoting HIPK-JNK signaling-mediated cell death and cell migration is evolutionarily conserved in its human ortholog ATXN2L.

## Discussions

Ataxin-2 (Atx2) and its human orthologs, ATXN2 and ATXN2L, have been implicated in a broad spectrum of diseases, ranging from neurodegenerative disorders such as spinocerebellar ataxia type 2 (SCA2) and amyotrophic lateral sclerosis (ALS) to Huntington’s disease (HD) and Parkinson’s disease (PD) [39]. Beyond the nervous system, ATXN2 has also been emerged as a risk factor in inflammatory diseases and multiple cancer types, including breast, colorectal, endometrial, lung, and gastric cancers [38, 40]. In contrast, ATXN2L shows weaker association with neurodegeneration but has been increasingly linked to tumorigenesis and cancer progression, such as GC and hepatocellular carcinoma (HCC) [41, 42]. Despite these associations, the molecular functions of Atx2 family proteins outside the nervous system have remained poorly defined. Here, we identify Atx2 as a conserved, positive regulator of JNK signaling and uncover a post-transcriptional mechanism by which Atx2 family proteins control Hipk-JNK pathway activity across development and disease contexts.

Our genetic analyses reveal that Atx2 functions downstream of the MAP3Ks dTak1 and Wnd and upstream of the MAP2K Hep in the canonical JNK cascade. Both gain- and loss-of-function approaches demonstrate that Atx2 acts as a potent amplifier of JNK signaling: ectopic expression triggers robust JNK activation, apoptosis, and tissue remodeling, whereas knockdown suppresses JNK-driven phenotypes, including Egr-induced eye degeneration and Ras^V12^/*lgl*^-^ tumor growth and invasion. These results position Atx2 as a key determinant of JNK pathway output. Given the multiple roles of JNK signaling in apoptosis, compensatory proliferation, and tumor progression [23, 31, 32], Atx2 likely fine-tunes the magnitude and persistence of JNK activation to shape distinct biological outcomes.

Mechanistically, we demonstrate that Atx2 regulates JNK signaling by stabilizing *hipk* mRNA, which encodes a well-established JNK activator [34]. RIP and GFP reporter assays confirmed that Atx2 directly binds the *hipk* transcript 3′UTR, thereby enhancing its stability and expression. Consistently, *hipk* knockdown abolished Atx2-induced JNK phosphorylation, apoptosis, and cell migration. These findings provide a mechanistic link between Atx2’s post-transcriptional activity and JNK pathway regulation, expanding the repertoire of *Atx2* mRNA targets previously identified by TRIBE-seq [33].

Beyond pathological signaling, Atx2 also proved indispensable for physiological JNK activity. During thorax closure, a morphogenetic process critically dependent on JNK [36, 37], *Atx2* depletion produced defects resembling JNK inhibition, while genetic enhancement of JNK signaling rescued these abnormalities. Similarly, in a Ras^V12^/*lgl*^*-*^ tumor model, *Atx2* knockdown suppressed tumor growth, invasion, and lethality, mirroring JNK inhibition. Notably, Atx2 overexpression selectively enhanced invasion without increasing tumor size, highlighting a specific role in regulating metastatic potential. These findings emphasize Atx2 as a central regulator of JNK signaling in both normal development and disease.

Importantly, our work demonstrates that this JNK-regulatory function of Atx2 is evolutionarily conserved in its human ortholog ATXN2L. Transgenic expression of either ATXN2 or ATXN2L in flies activated JNK signaling and induced JNK-dependent apoptosis, indicating partial conservation at the organismal level. However, in human HeLa cells, only ATXN2L retained the ability to activate JNK signaling, induce cell death, and promote cell migration and invasion, all of which were dependent on HIPK expression. Consistently, depletion of *ATXN2L*, but not *ATXN2*, reduced *HIPK* mRNA levels and attenuated JNK phosphorylation. These findings indicate a functional divergence between ATXN2 and ATXN2L in mammalian systems and suggest that ATXN2L has retained the dominant ancestral role in Hipk–JNK regulation.

A notable structural distinction between ATXN2 and ATXN2L is the presence of a polyglutamine (polyQ) tract in ATXN2, which is absent in ATXN2L [41]. PolyQ expansion in ATXN2 is a hallmark of neurodegenerative disease and is often associated with loss or alteration of normal protein function [43–45]. Consistent with this notion, a polyQ-expanded ATXN2 variant (ATXN2^117Q^) failed to activate JNK signaling in our assays, suggesting that polyQ expansion disrupts the physiological role of ATXN2 family proteins in stress-activated signaling pathways. These findings further support the idea that JNK regulation by Atx2 family proteins is independent of polyQ-mediated toxicity and instead reflects an ancestral, conserved function.

Our work raises several important questions for future investigation. First, how does Atx2-mediated stabilization of *hipk* mRNA integrate with other transcriptional and post-translational regulators of the JNK pathway? Atx2 may function as a critical post-transcriptional node that fine-tunes JNK signaling dynamics in response to developmental cues or cellular stress. Second, given Atx2’s established role in stress granule assembly, it will be important to determine whether *hipk* mRNA is dynamically regulated within stress granules under conditions of oxidative or proteotoxic stress, providing a rapid mechanism to modulate JNK activity and cell fate decisions. Third, the conserved role of ATXN2L in HIPK-JNK activation provides a direct mechanistic link to its association with cancer progression. Elevated JNK signaling has been implicated in tumor invasion, metastasis, and therapy resistance in specific contexts, raising the possibility that dysregulated ATXN2L expression contributes to oncogenesis through hyperactivation of this pathway.

In summary, this study identifies Atx2 as a conserved post-transcriptional regulator of JNK signaling via stabilization of *hipk* mRNA. By linking Atx2 family proteins to JNK pathway control, we broaden their functional relevance from neurodegeneration to developmental morphogenesis and tumorigenesis. These findings raise the possibility that dysregulated Atx2 family proteins contributes to disease by perturbing stress-activated signaling. Future studies in mammalian models will be critical to determine whether targeting the Atx2-Hipk axis could provide therapeutic benefit in JNK-driven cancers.

## Materials and methods

### *Drosophila* genetics and stocks

All flies were raised on standard cornmeal-sugar-yeast media at 25 °C unless otherwise noted. For experiments using *ptc*-Gal4, crosses were shifted to 29 °C to enhance Gal4 activity (Fig. 5, Fig. S5). For larva experiments, both male and female files were used. For adult experiments, female files were used.

The following *Drosophila* lines have been described previously: *GMR*-Gal4, *sev*-Gal4, *ptc*-Gal4, *nub*-Gal4, *pnr*-Gal4, *UAS*-GFP, *UAS*-Egr, *UAS*-dTak1, *UAS*-Hep^CA^ [46], *puc*^*E69*^ [47], *TRE*-RFP [27], *UAS*-Puc [48], *UAS*-P35, *UAS*-*dTak1-IR* [49], and *UAS*-*bsk-IR*. The *hipk* mutant line was kindly provided by Dr. Renjie Jiao [34].

Additional lines were obtained from the following sources: *UAS*-LacZ (#3956), *UAS-GFP-IR* (#9330), *UAS*-Dcr2 (#24651), *UAS-Atx2-IR* (#36114), and *UAS*-ATXN2.117Q (#68395) from the Bloomington *Drosophila* Stock Center; *UAS-Atx2-IR* (#108843, #34955), *UAS-hep-IR* (#V47507), and *UAS-hipk-IR* (#108254) from the Vienna *Drosophila RNAi* Center; and UAS-Atx2 (#001031) from FlyORF [50]. *UAS*-ATXN2 and *UAS*-ATXN2L transgenic flies were generated by PCR amplification of full-length cDNAs followed by subcloning into the pUAST vector. All fly genotypes utilized in this study are listed in Table S1.

The Ras^V12^/*lgl*^*-*^ tumor model was generated using the following genotypes: *y w*, *ey*-Flp; *tub*-Gal80 *FRT40A*; *act* > y + >Gal4 *UAS*-GFP and *w*; *lgl*^*4*^ *FRT40A UAS*-Ras^V12^ [51]. Cephalic complexes were dissected at 8–9 days after egg laying (AEL).

### Acridine orange (AO) staining

Eye discs were dissected from third instar larvae in 0.1% PBST (PBS with 0.1% Tween-20), incubated in 10⁻² mM AO for 30 s at room temperature, and rinsed once in PBST before mounting [52].

### Immunohistochemistry

Antibody staining was performed by standard procedures for imaginal discs [53]. The following antibodies were used: rabbit anti-active Caspase-3 (Cell Signaling Technology (CST), Massachusetts, USA, #Asp175, 1:400), rabbit anti-phospho-JNK (p-JNK, Calbiochem, California, USA, #559309, 1:200), and goat anti-rabbit CY3 secondary antibody (Life Technologies, Shanghai, China, #A10521, 1:1000), β-Actin (D6A8) Rabbit mAb (Cell Signaling Technology, 8457S); HA-Tag (C29F4) Rabbit mAb (Cell Signaling Technology, 3724S); GFP (D5.1) Rabbit Monoclonal Antibody (Cell Signaling Technology, 2956S); p-JNK (Thr183/Tyr185) Antibody (Cell Signaling Technology, 9251S); JNK Antibody (Cell Signaling Technology, 9252S).

### X-gal staining

Wing discs were dissected from third instar larvae in PBST (1× PBS, pH 7.0, 0.1% Triton X-100), and stained for β-galactosidase activity as previously described [54].

### Cell culture and treatment

HeLa cells were cultured in DMEM (Gibco, California, USA) with 10% FBS (Corning, New York, USA) and 1% penicillin–streptomycin (Procell, Wuhan, China) at 37 °C in a humidified 5% CO₂ incubator. *Drosophila* S2 cells were maintained in Schneider’s medium (Gibco, California, USA) supplemented with 10% FBS (TransGen, Beijing, China) and 1% penicillin–streptomycin at 26 °C.

Cells were seeded 24 h prior to transfection. Plasmid DNA was transfected into mammalian cells using Lipofectamine 3000 (Invitrogen, California, USA) and into S2 cells using X-tremeGENE HP DNA Transfection Reagent (Roche, Basel, Switzerland). siRNAs were transfected into HeLa cells using Lipofectamine RNAiMAX (Invitrogen, California, USA). Cell death was detected by PI staining according to the manufacturer’s instructions (Beyotime, Shanghai, China).

The human HeLa cell was purchased from Procell. STR profiling was performed by the vendor upon purchase (August 2022) to confirm the cell line’s identity. Routine mycoplasma testing using the One-Step Mycoplasma Detection Kit (Yeasen) was conducted, with the last negative test result obtained in October 2025.

### Plasmid construction

Full-length cDNA of human ATXN2 and ATXN2L were amplified from the cDNA library of 293T cells or HeLa cells. Full-length cDNA of *Drosophila Atx2* and *hipk* 3’UTR were amplified from the cDNA library of S2 cells or Kc cells. PCR products were digested with appropriate restriction enzymes (New England Biolabs, Massachusetts, USA) and cloned into pCMV/pcDNA3.1 or pUAST vectors. Primer sequences are listed below:

*ATXN2*:

5′-GCT AAG CTT ATG AGG ATG GTT CAT ATA CTT ACA TCA GTT-3′

5′-TCC TCT AGA TTA CAA CTG CTG TTG GTG GTG G-3′

*ATXN2L*:

5′-CAG GAA TTC ATG TTG AAG CCT CAG CCG CTA CAA C-3′

5′-AGT TCT AGA TTA CTC CCC ACC CAG AAC CCG-3′

*Atx2:*

5′-ATA GAT CTA TGA ACA ACA ATA GCA AGC GGA AAA CC-3′

5′-ACC CTC GAG TCA CTG TGG CTG ATG CTG CTG CAT C-3′

*Hipk* 3′ UTR:

5′-CAT AGA TCT GCG GCC GCA TAA ACC GAC GAT GGG AGT TGA CGG-3′

5′-AGA GGT ACC GAT ACA CGC ATA TTG CGC ACA TTA AAT C-3′

### Quantitative PCR (qPCR)

RNA was extracted using NucleoZOL (MACHEREY-NAGEL, Germany) and reverse-transcribed with the All-in-One First-Strand cDNA Synthesis Kit (TransGen, Beijing, China). qPCR was performed using SYBR Green Master Mix (Yeasen, Shanghai, China) on a Stratagene Mx3000P system (Agilent, California, USA), with 40 amplification cycles. Primer sequences are listed below:

Human *GAPDH* (internal control):

5′-GGA GTC AAC GGA TTT GGT CGT-3′

5′-GAT CTC GCT CCT GGA AGA TGG T-3′

*HIPK1*:

5′-TCT CAG TGC CGG AAC AAA AAC-3′

5′-CCC TCC AGG TCT GTA GAC ATA TT-3′

*HIPK2*:

5′-AAT AGA GCC GAG TTC CAA CTG G-3′

5′-GTC TGC TCG TAA GGT AGG CTT-3′

*HIPK3*:

5′-TCA CAA GTC TTG GTC TAC CCA-3′

5′-CAC ATA GGT CCG TGG ATA GTT TC-3′

*ATXN2*:

5′-TTG ATG CCG CAC ATG AGA AAA-3′

5′-CGC CAT TCA CTT TAG CAC TGA T-3′

*ATXN2L*:

5′-GAA CTA GCC GTG GAT GCT GTG-3′

5′-GCT GAA TCG GTG AAC TTG TCT T-3′

*Drosophila rp49* (internal control):

5′-AGA TCG TGA AGA AGC GCA CCA AG-3′

5′-CAC CAG GAA CTT CTT GAA TCC GG-3′

*GFP*:

5′-CGA CCA CTA CCA GCA GAA CAC C-3′

5′-CGA ACT CCA GCA GGA CCA TGT-3′

*Hipk:*

5′-AGT GCA GCA GGA CTC TTC AGC A-3′

5′-CGA TAG GGA ATA GAG CAC CTC GT-3′

*Atx2:*

5′-CAA GGA CTT TGA CTC GCA ATA CG-3′

5′-ACC GTT GCA CTT GTC AGA AAT G-3′

### Immunoblotting and RNA immunoprecipitation (RIP)

Cells or tissues were washed in ice-cold PBS and lysed in RIPA buffer (Beyotime, Shanghai, China) containing protease inhibitors (Yeasen, Shanghai, China) and PMSF (Beyotime, Shanghai, China) on ice for 10 min. Proteins were separated on 10% SDS-PAGE gels (Vazyme, Jiangsu, China) and transferred to PVDF membranes for immunoblotting.

Primary antibodies: Anti-JNK (CST, Massachusetts, USA, 1:2000); Anti-p-JNK (CST, Massachusetts, USA, 1:2000); Anti-HA (CST, Massachusetts, USA, 1:4000); and Anti-β-actin (CST, Massachusetts, USA, 1:4000)

Secondary antibodies were HRP-conjugated (Abways, Shanghai, China), and signals were visualized using SuperPico ECL Chemiluminescence Kit (Vazyme, Jiangsu, China). RNA-binding protein immunoprecipitation was performed using the Magna RIP™ Kit (Millipore, Massachusetts, USA). Band intensities were quantified using ImageJ.

### RNA interference

*siRNAs* targeting human *HIPK1*, *HIPK2*, and *HIPK3* were synthesized by Huagene. Sequences are listed below:

*si-HIPK1-1:*

Sense: 5′-GCC AUC AUC UAC GAA GUA UTT-3′

Antisense: 5′-CGG UAG UAG AUG UUC UAU ATT-3′

*si-HIPK1-2:*

Sense: 5′-CCU CAU AUU CCU GGA GAA ATT-3′

Antisense: 5′-GGA GUA UAA GGA CCU CUU UTT-3′

*si-HIPK2-1:*

Sense: 5′-GCA UCA AGC UGA AGA ACA UTT-3′

Antisense: 5′-CGU AGU UCG ACU UCU UGU ATT-3′

*si-HIPK2-2:*

Sense: 5′-CUG GAU AAA UUC CUG CUA ATT-3′

Antisense: 5′-GAC CUA UUU AAG GAC GAU UTT-3′

*si-HIPK3-1:*

Sense: 5′-GGA UGA UGC UGA AAG UAU ATT-3′

Antisense: 5′-CCU ACU ACG ACU UUC AUA UTT-3′

*si-HIPK3-2:*

Sense: 5′-UUA CAU UCU GUC CAC UAA ATT-3′

Antisense: 5′-AAU GUA AGA CAG GUG UUU UTT-3′

*si-ATXN2-1:*

Sense: 5′-UCA GAC UUU GUU GUG GUA CAG UUU A-3′

Antisense: 5′-UAA ACU GUA CCA CAA CAA AGU CUG A-3′

*si-ATXN2-2:*

Sense: 5′-CCA AGC UGG UAU UAU UCC AAC UGA A-3′

Antisense: 5′-UUC AGU UGG AAU AAU ACC AGC UUG G-3′

*si-ATXN2L-1:*

Sense: 5′-CCU GUG UUU GAA GGC GUC UAC AAC A-3′

Antisense: 5′-UGU UGU AGA CGC CUU CAA ACA CAG G-3′

*si-ATXN2L-2:*

Sense: 5′-UCA GAU CCU GGA GUG GGC UCC AUU U-3′

Antisense: 5′-AAA UGG AGC CCA CUC CAG GAU CUG A-3′

Knockdown efficiency was validated by qPCR.

### Cell migration and invasion

Matrigel matrix (Corning) was mixed with Dulbeccos’s Modified Eagle Medium (DMEM) (1:8) and 60 µL of the mixture was laid over the transwell insert (LABSELECT) placed on top of a 24-well plate. The mix was left undisturbed 3 h in a 37 °C CO2 incubator. A total of 20,000 cells in 100 µL medium were added on top of the insert and an additional 500 µL of medium containing 10% FBS was added into the plate well below the insert (Matrigel matrix was not added over the transwell insert in cell migration assay). The cells were incubated for 48 h in a CO2 incubator. Then, the supernatant and the Matrigel matrix in the insert were removed and the invading cells on the membrane of the insert were stained with 0.1% Crystal Violet (Yeasen) and counted using ImageJ.

### Statistical analysis

For all statistical analyses presented in the figures, the choice of statistical test for every analysis was predetermined based on the experimental design, the type of data (continuous or categorical), and whether the data met the underlying assumptions of parametric tests. Prior to conducting parametric tests (e.g., *t*-test, ANOVA), we assessed whether the data met the assumptions. Normality was tested using the Shapiro-Wilk test (*n* < 50). Homogeneity of variances was assessed using Levene’s test. For normally distributed data, *t*-tests or ANOVA were used. Non-normally distributed data were analyzed using the Mann–Whitney test. All statistical analyses were performed using GraphPad Prism 8.0. Data were expressed as scatter plots or bar graphs. Data are mean ± SEM. Statistical significance was defined as *p* < 0.05. Asterisks indicate significance as follows: *p* < 0.05 (*), *p* < 0.01 (**), *p* < 0.001 (***), and *p* < 0.0001 (****).

Our statistical analyses were rigorously performed as follows:

For morphology experiment: Sample sizes were selected based on prior experience with these assays and published studies in the field, which consistently show that the phenotypes produced by genetic manipulations of this type are robust and reproducible. Flies were selected without regard to phenotype. Samples were mounted and imaged in randomized order and all genotypes were processed in parallel, and objective quantitative measurements were used for all analyses. Eye, wing, and tumor sizes, as well as cell death counts, were measured using Adobe Photoshop 2018. p-JNK fluorescence intensity was quantified using ImageJ.

For qPCR: Gene expression was normalized to the internal reference genes (*GAPDH* in mammalian cells, *rp49* in *Drosophila*), yielding ΔCt values. Comparisons between two groups employed an unpaired two-tailed *t*-test, while multiple comparisons used ANOVA with Tukey’s post hoc test.

For Western Blot: Protein band intensities were quantified by ImageJ and normalized to the loading control (β-actin). Statistical comparisons were then conducted using the same parametric framework (*t*-test or ANOVA) as described for qPCR.

For PI and Crystal Violet Staining: Cell number was quantified by ImageJ, statistical comparisons were then conducted using one-way ANOVA with Tukey’s post hoc test.

## Supplementary information

Supplementary Figures

Supplementary Table 1

Supplemental Material-Western original Files

## Data Availability

The data that support the finding of this study are available from the corresponding author upon reasonable request.
