# Supplementary material for: Conserved role of Atx2 in JNK pathway activation
Source: Cell Death Dis. 2026 May 2;17(1):585. doi: 10.1038/s41419-026-08797-9 (PMC13279927; doi:10.1038/s41419-026-08797-9)
Supplement: Supplementary file 1 — Supplementary Figures [file 41419_2026_8797_MOESM1_ESM.docx]

**
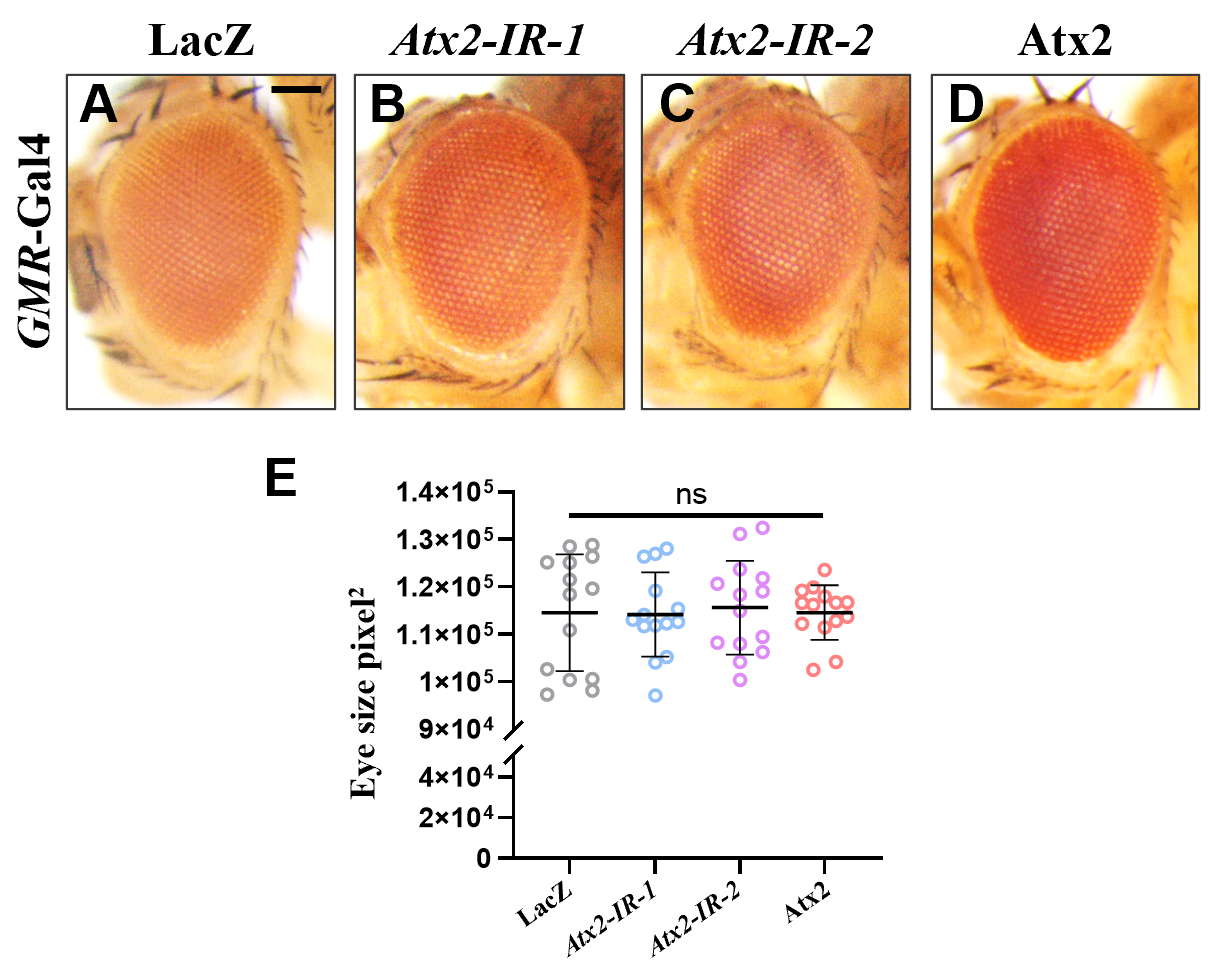
**

**Figure S1. Overexpression or depletion of *Atx2* driven by *GMR*-Gal4 does not affect eye size.**

Light micrographs of adult *Drosophila* eyes. Compared with the *GMR*>LacZ control (A), downregulation of *Atx2* did not affect eye size (B, C). (D) Overexpression of Atx2 did not alter eye size. (E) Quantification of adult eye size (*n*=14 per group). Statistical significance was determined using One-way ANOVA; n.s., not significant. Scale bars: 100 μm (A-D).

**
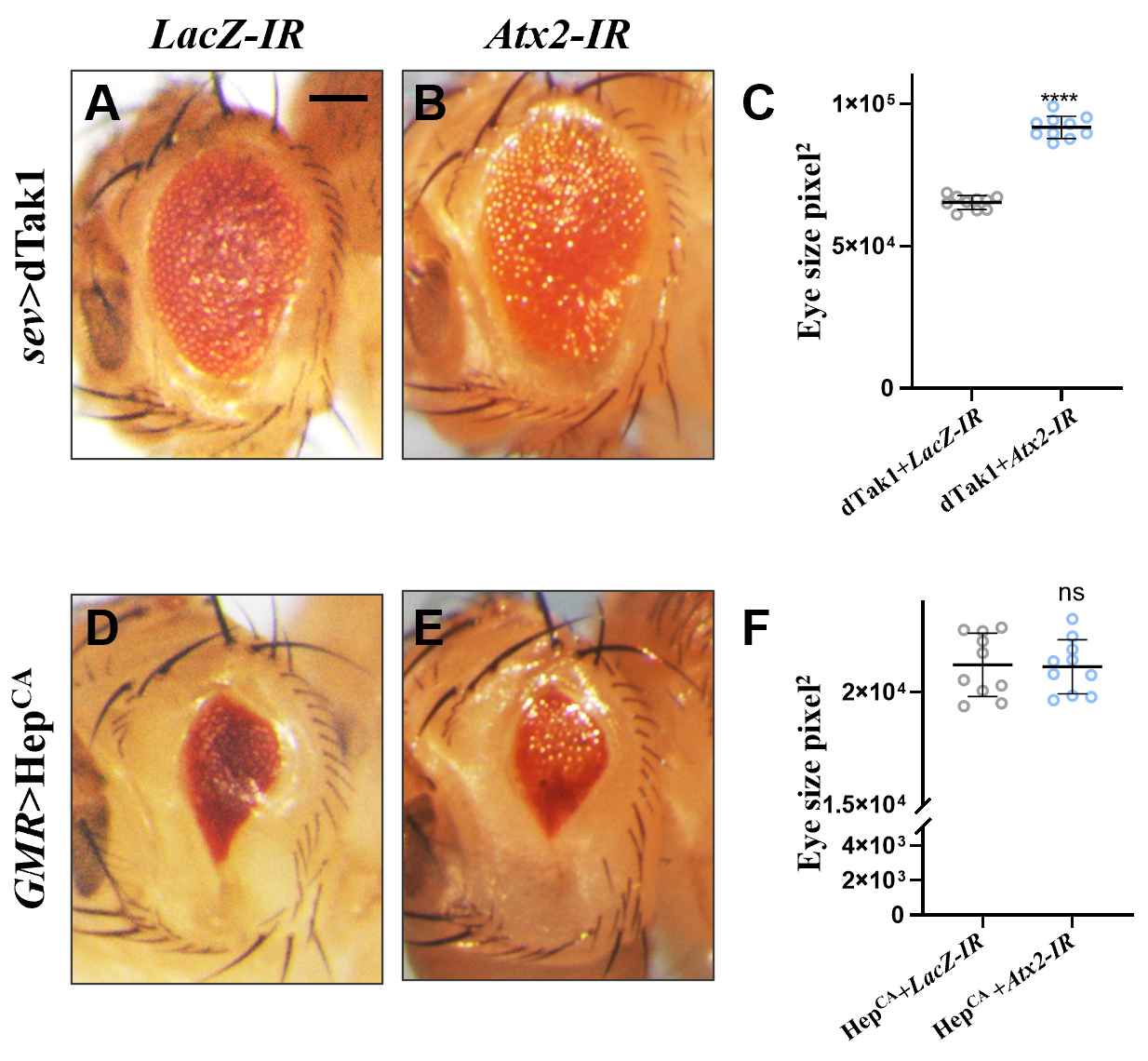
**

**Figure S2. Atx2 functions downstream of dTak1 and upstream of, or parallel to, Hep.**

Light micrographs of adult *Drosophila* eyes. Overexpression of dTak1 driven by *sev*-Gal4 produced a small, rough-eye phenotype (A), which was rescued by *Atx2* depletion (B). By contrast, expression of a constitutively active Hep (Hep^CA^) driven by *GMR*-Gal4 also reduced eye size (D), but this phenotype was not suppressed by *Atx2* loss (E). Quantification of adult eye size is shown in (C, F) (*n*=10 per group). Statistical significance was determined by unpaired two-tailed t-test: ****p < 0.0001; n.s., not significant. Scale bars: 100 μm (A-B, D-E).


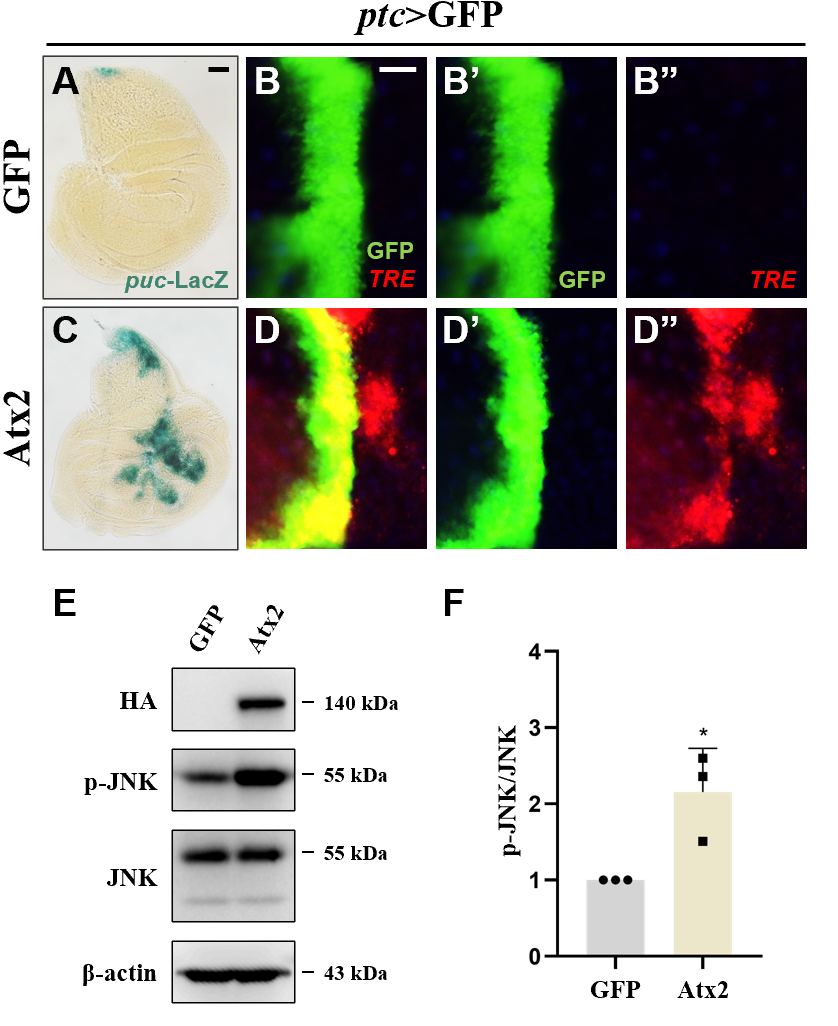


**Figure S3. Atx2 activates JNK signaling.**

(A, C) Light micrographs of third instar larval wing discs stained with X-gal (*n*=5 per group). Compared with controls (A), Atx2 overexpression strongly activated *puc*-LacZ expression along the A/P boundary (C). (B-B″, D-D″) Fluorescence images of wing discs. *ptc*-expressing cells were marked by GFP. Compared with controls (B-B″), ectopic Atx2 expression induced activation of the *TRE*-RFP reporter (D-D″). (E) Immunoblot analysis of phosphorylated JNK (p-JNK) and total JNK from third instar larval tissues overexpressing Atx2 under *hs*-Gal4. Lysates were collected in RIPA buffer 6 h after heat shock. (F) Quantification of p-JNK levels (*n*=3 per group). Statistical significance was determined by unpaired two-tailed t-test: p < 0.05. Scale bars: 100 μm (A, C); 25 μm (B, D).

**
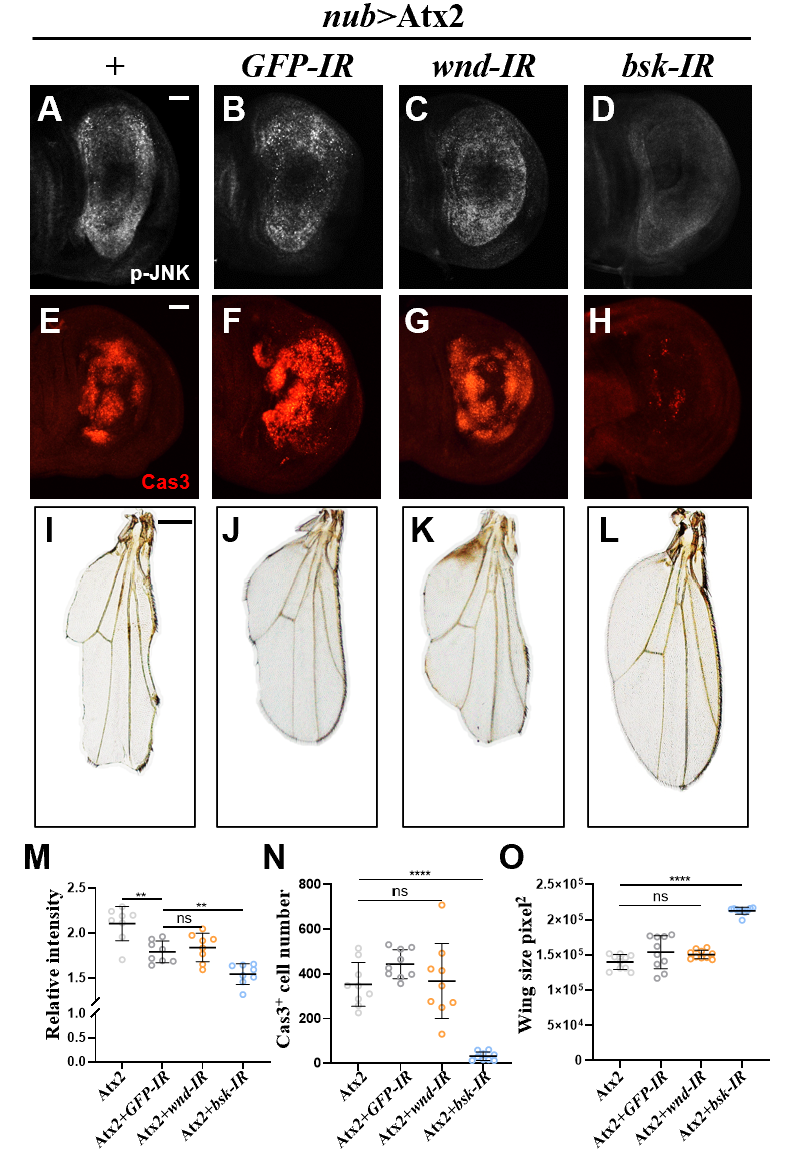
**

**Figure S4. Atx2 acts downstream of Wnd and upstream of Bsk.**

(A-D) Fluorescence micrographs of wing discs stained for phosphorylated JNK (p-JNK). Atx2 overexpression induced p-JNK accumulation (A), as a negative control, *GFP RNAi* slightly suppressed the relative intensity of p-JNK staining (B). Compare with control, depletion of *wnd* had no effect on p-JNK accumulation caused by Atx2 overexpression (C), whereas *bsk RNAi* significantly suppressed p-JNK accumulation (D). (E-H) Fluorescence images of cleaved Caspase-3 staining. (E) Overexpression of Atx2 caused Caspase-3 activation, which was unaffected by *GFP RNAi* (F) or *wnd RNAi* (G), but suppressed by *bsk RNAi* (H). (I-L) Light micrographs of adult wings. (I) *nub*>Atx2 induced small wing phenotype, which was not rescued by *GFP RNAi* (J) or *wnd RNAi* (K), but was suppressed by *bsk RNAi* (L). (M-O) Quantification of p-JNK intensity (*n*=8 per group) (M), number of Caspase-3-positive cells (*n*=9 per group) (N), and adult wing size (*n*=10 per group) (O). Data are mean ± SEM. One-way ANOVA: ****p < 0.0001, **p<0.01; n.s., not significant. Scale bars: 50 μm (A-H), 250 μm (I-L).


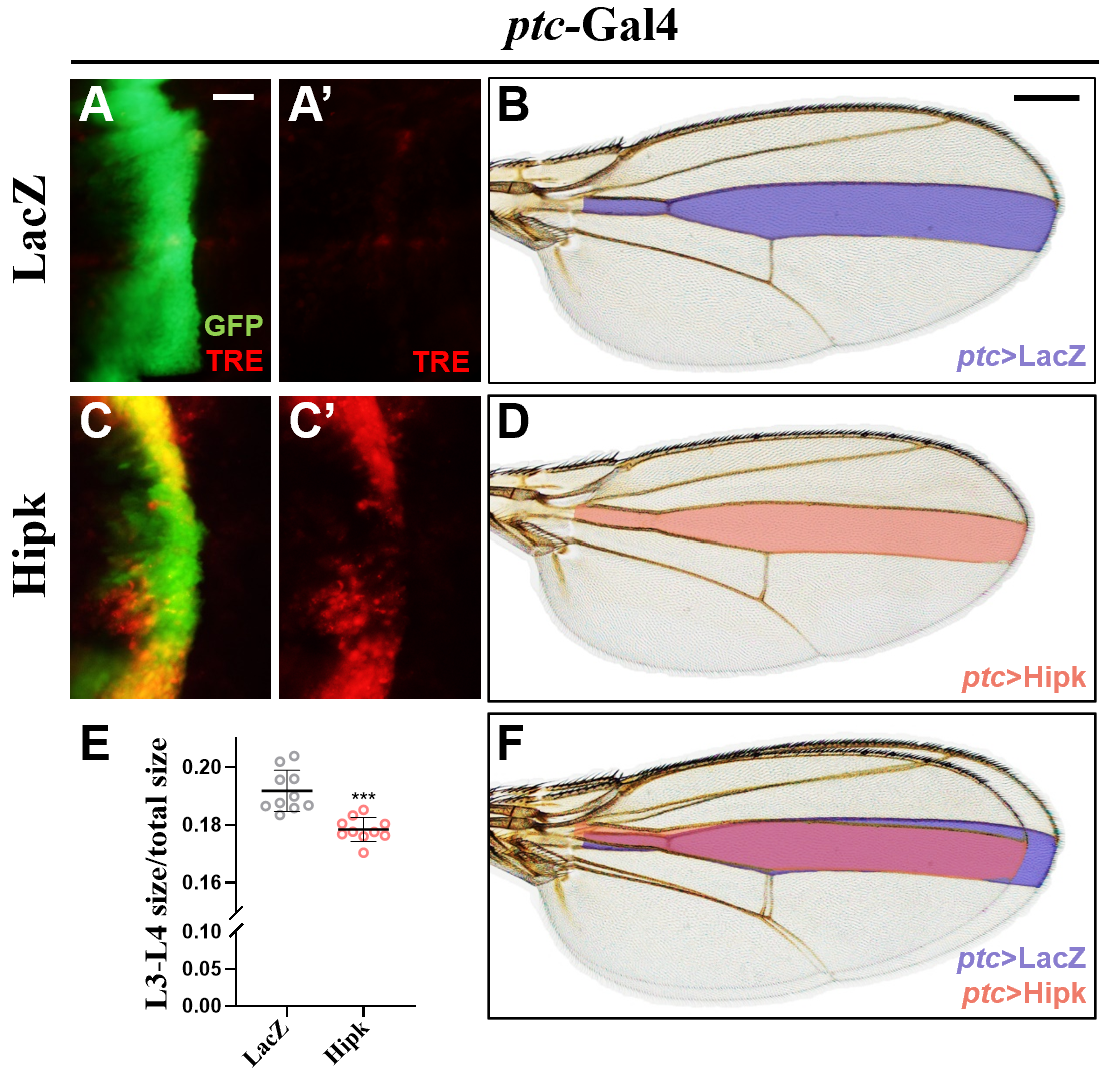


**Figure S5. Hipk overexpression activates JNK signaling.**

(A, A’) Control wing disc expressing the JNK reporter TRE-RFP shows baseline fluorescence. (B) Control adult wing highlighting the normal L3-L4 intervein region (outlined in blue). (C, C’) Hipk overexpression (*ptc*>Hipk) strongly activates the JNK pathway, as indicated by elevated TRE-RFP expression. (D) Adult wing with Hipk overexpression displays a pronounced reduction in the L3-L4 intervein area (outlined in pink). (E) Quantification of the relative L3-L4 intervein size, normalized to total wing area (*n*=10 per group). (F) Overlay of control (*ptc*>LacZ) and Hipk-overexpressing (*ptc*>Hipk) wings, illustrating the decrease in intervein territory upon Hipk induction. Statistical significance was determined by unpaired two tailed T-test: ***p < 0.001. Scale bars: 50 μm (A, C), 250 μm (B, D).


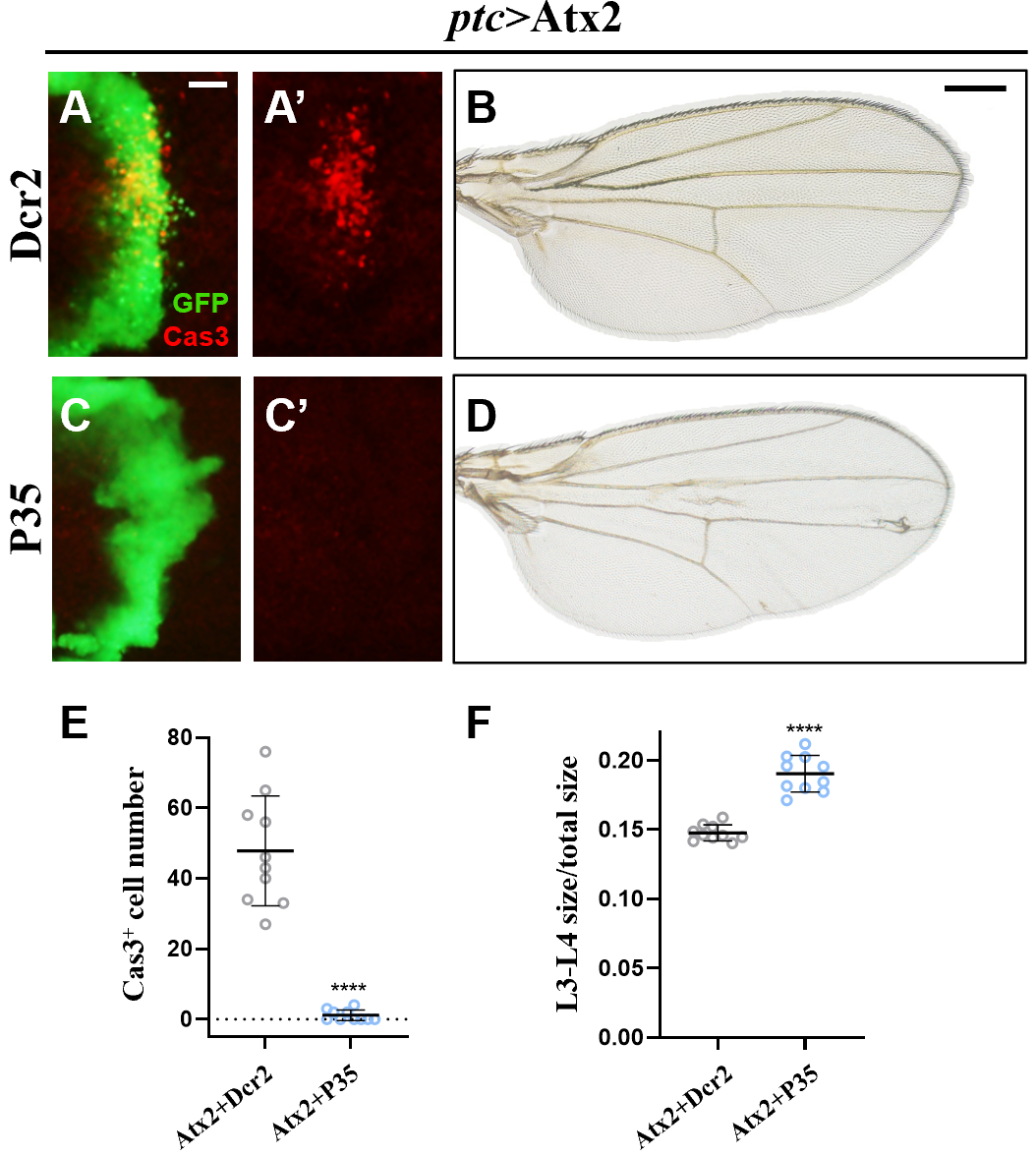


**Figure S6. The Atx2-induced wing phenotype** **is a direct consequence of apoptosis.**

(A-B) As a negative control, *UAS*-Dcr2 did not suppress Caspase activation (A-A’) or the reduced L3-L4 intervein region (B) induced by *ptc*>Atx2. (C-C’) overexpression of the anti-apoptotic protein P35 significantly decreased Caspase activation and rescued the L3-L4 wing area (D). Quantification of the number of Caspase-3-positive cells (*n*=10 per group) (E) and the ratio of L3-L4 size to total wing size (*n*=10 per group) (F). Statistical significance was determined by unpaired two tailed T-test: ****p < 0.0001. Scale bars: 50 μm (A, C), 250 μm (B, D).


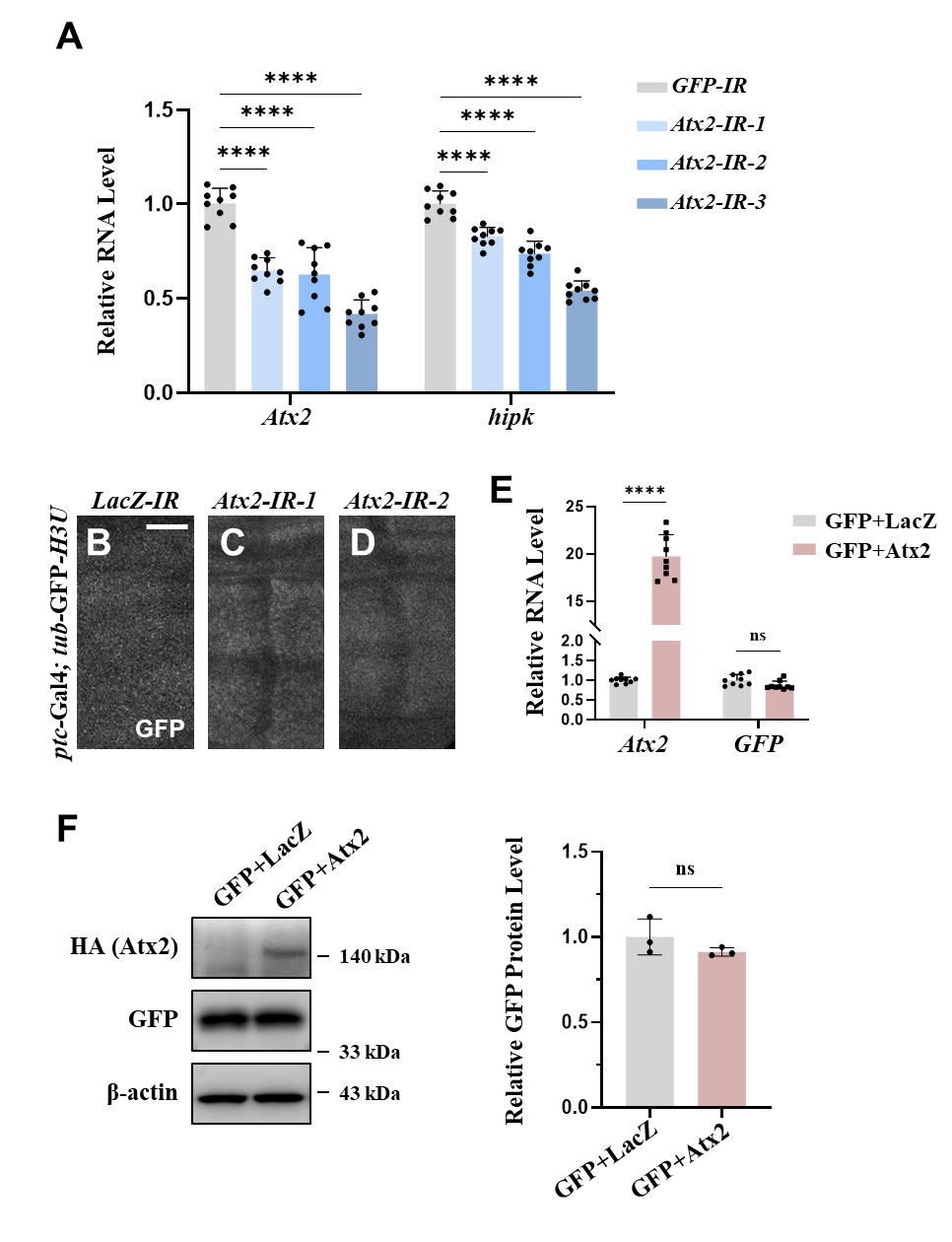


**Figure S7. Loss of *Atx2* reduces Hipk expression and Atx2 overexpression does not affect GFP expression levels.**

(A) RT-qPCR analysis showing *Atx2* knockdown reduced endogenous *hipk* mRNA levels. *Atx2-IR* was driven by *hs*-Gal4, and 3^rd^ instar larval tissues were lysed in NucleoZOL 18 h after heat shock to extract RNA. (B-D) Wing discs expressing *tub*-GFP-*H3U* (*n*=5 per group). Compared with control (B), deletion of *Atx2* driven by *ptc*-Gal4 decreased GFP reporter intensity (C, D). (E) RT-qPCR analysis shows that overexpression of Atx2 in an *act*>GFP background had no effect on GFP mRNA levels. (F) Immunoblot analysis showing that Atx2 overexpression in an *act*>GFP background did not affect GFP protein levels in one-third of third-instar larval tissues. Data are presented as mean ± SEM. Statistical significance was determined using two-way ANOVA or unpaired two-tailed t-test (*n*=3 per group): ****p < 0.0001; n.s., not significant. Scale bar: 50 μm (B-D).


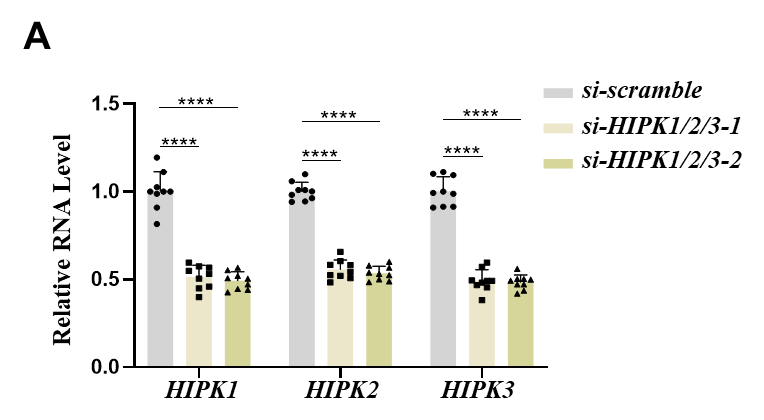


**Figure S8. Knockdown efficiency of *HIPK* RNAi.**

(A) Validation of *HIPK1/2/3* knockdown efficiency in HeLa cells using two independent siRNA combinations. Cells were lysed in NucleoZOL to extract RNA. Data are presented as mean ± SEM. Statistical significance was determined using two-way ANOVA (*n*=3 per group): ****p < 0.0001.


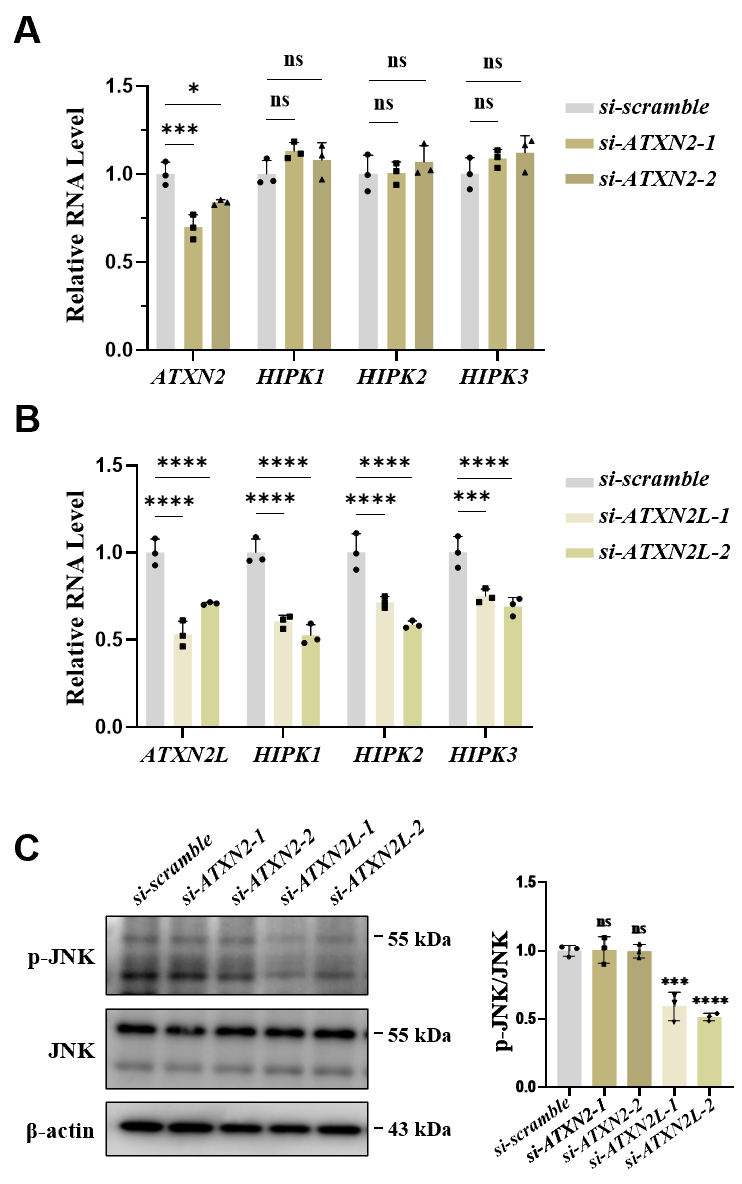


**Figure S9. Knockdown of *ATXN2L*, but not *ATXN2*, decreases *HIPK* RNA levels and JNK phosphorylation in HeLa cells.**

(A, B) RT-qPCR analysis following knockdown of *ATXN2* or *ATXN2L* in HeLa cells. Cells were lysed in NucleoZOL for RNA extraction. Knockdown of *ATXN2L*, but not *ATXN2*, reduced *HIPK1/2/3* mRNA levels. (C) Immunoblot analysis following knockdown of *ATXN2* or *ATXN2L* in HeLa cells. Cells were lysed in RIPA buffer for protein extraction. Knockdown of *ATXN2L*, but not *ATXN2*, reduced relative JNK phosphorylation, as indicated by the p-JNK/JNK ratio. Data are presented as mean ± SEM. Statistical significance was determined using two-way ANOVA (*n*=3 per group): ****p < 0.0001, ***p<0.001; n.s., not significant.


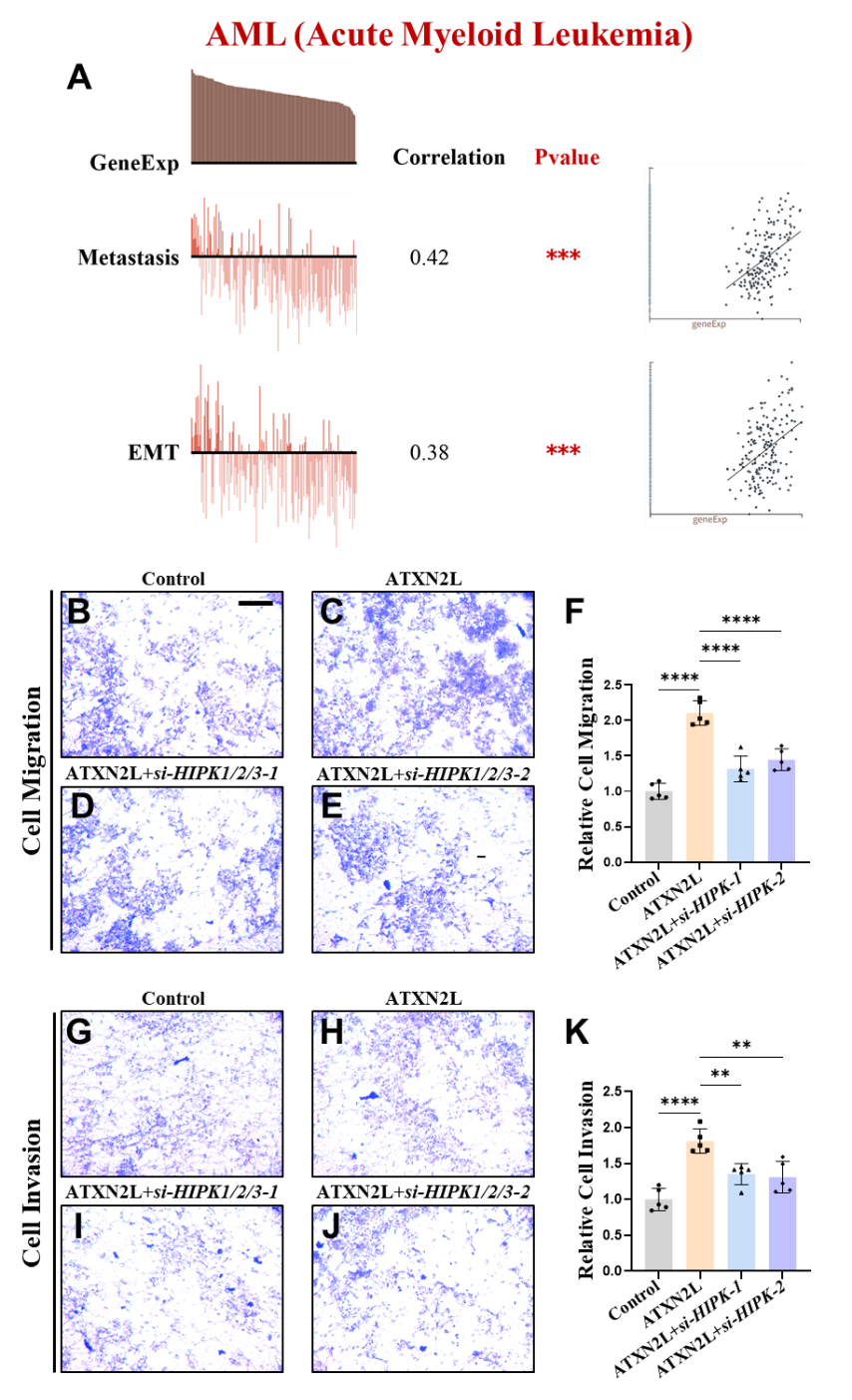


**Figure S10. ATXN2L is positively correlated with EMT in AML and induces cell migration and invasion via HIPK in HeLa cells.**

(A) Relevance of ATXN2L across several functional states, especially EMT and metastasis, in the CancerSEA database. (B-E, G-J) Crystal violet staining images. (B-E) Transwell migration assay. Compared with control (B), overexpression of ATXN2L promoted cell migration in HeLa cells (C), which was suppressed by deletion of *HIPK* (D-E). (G-J) Transwell invasion assay. Compared with control (G), ATXN2L overexpression induced cell invasion in HeLa cells (H), which was suppressed by *HIPK* deletion (I-J). (F, K) Statistical analysis of cell migration and invasion (*n*=5 per group). Cell numbers were quantified using ImageJ. Data are presented as mean ± SEM. One-way ANOVA: ****p < 0.0001, **p < 0.01. Scale bars: 50 μm (B-E, G-J).
