# Supplementary material for: Conserved role of Atx2 in JNK pathway activation
Source: Cell Death Dis. 2026 May 2;17(1):585. doi: 10.1038/s41419-026-08797-9 (PMC13279927; doi:10.1038/s41419-026-08797-9)
Supplement: Supplementary file 3 — Supplemental Material-Western original Files [file 41419_2026_8797_MOESM3_ESM.docx]

**Fig. 8J**

β-actin HA (ATXN2/ATXN2L)


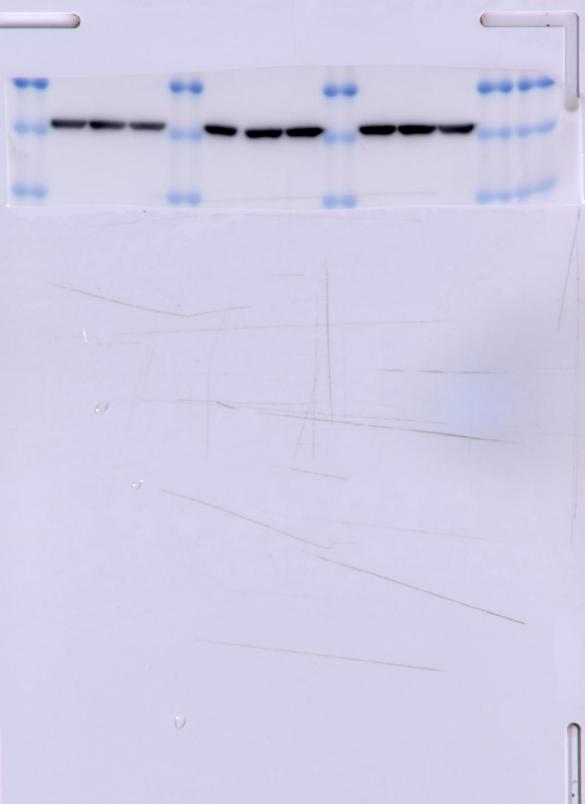

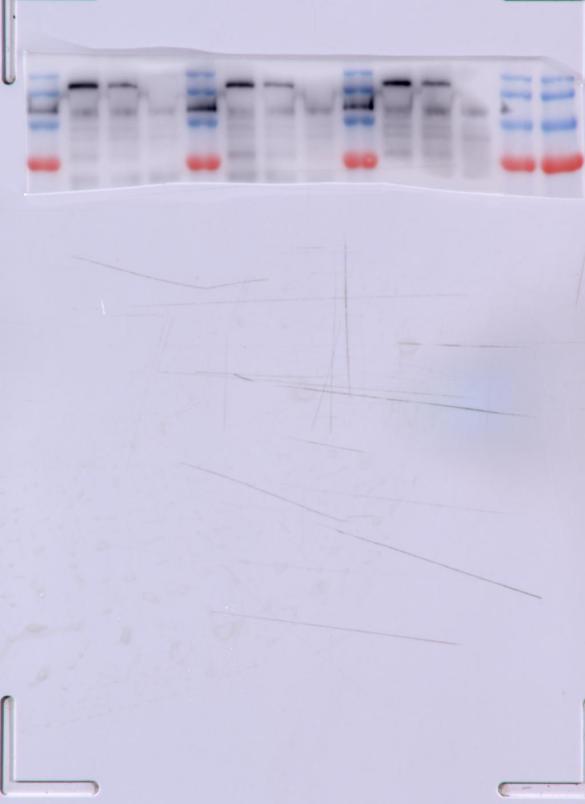


JNK pJNK


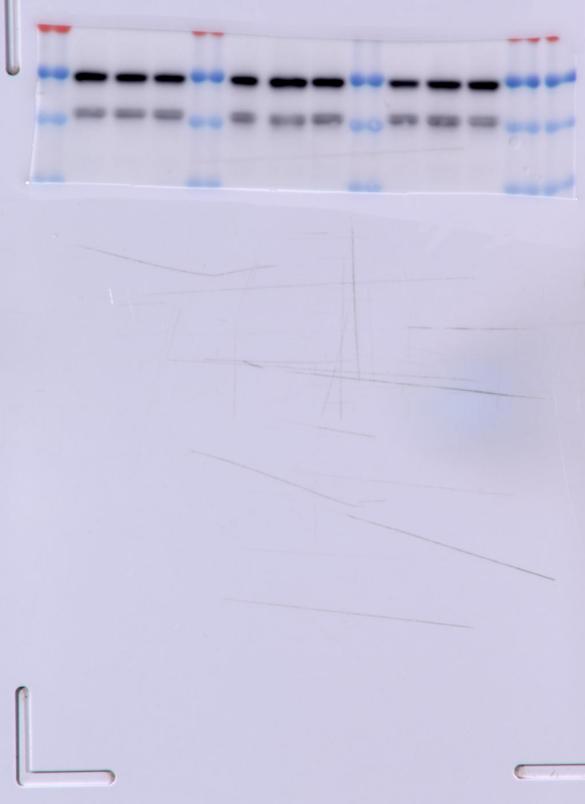

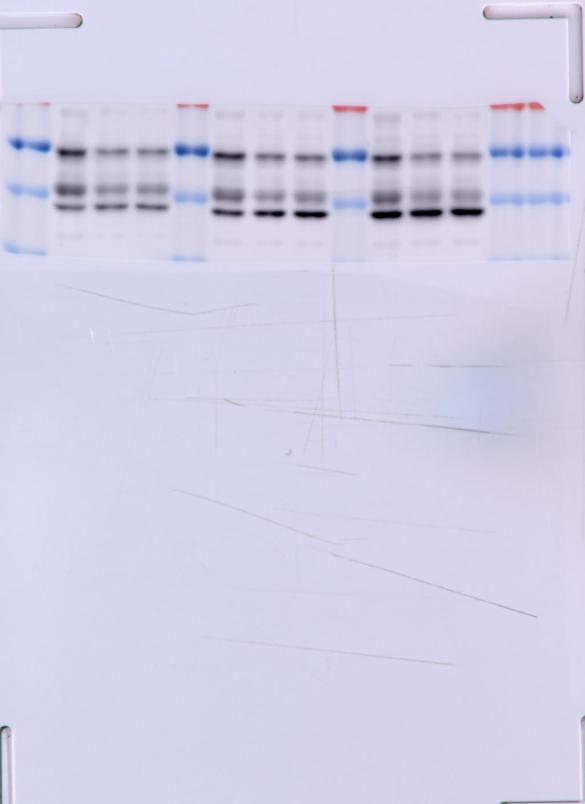


**Fig. 8K**

β-actin JNK


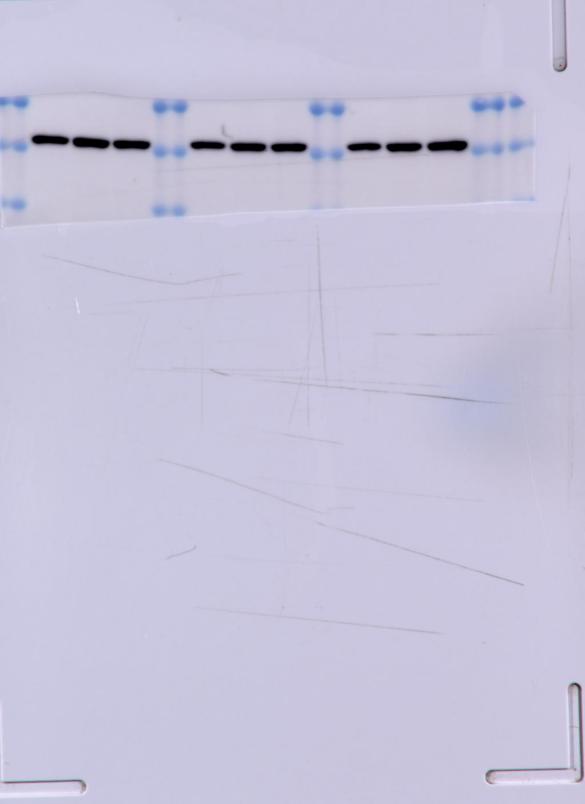

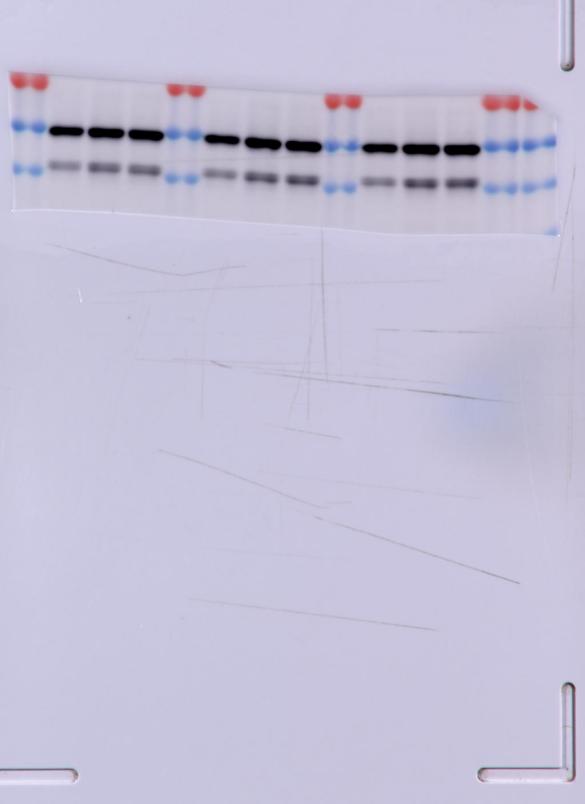


pJNK


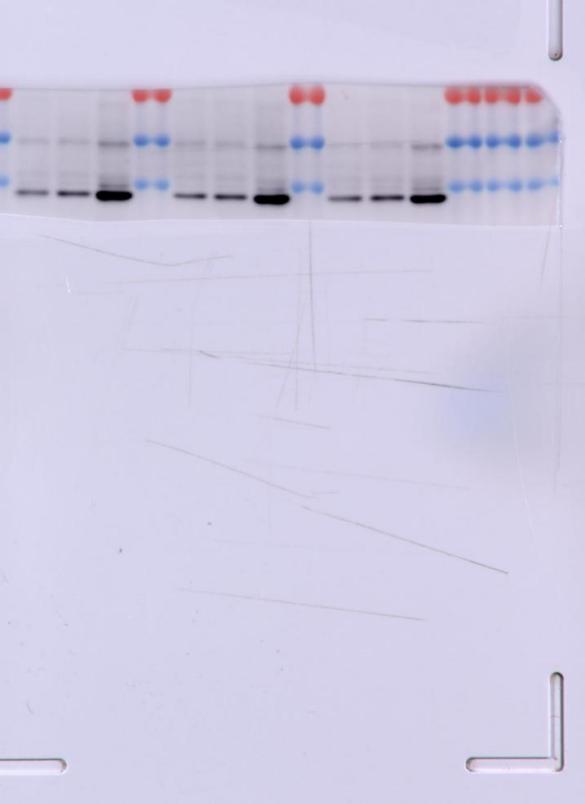


**Fig. S3E**

β-actin HA (Atx2)


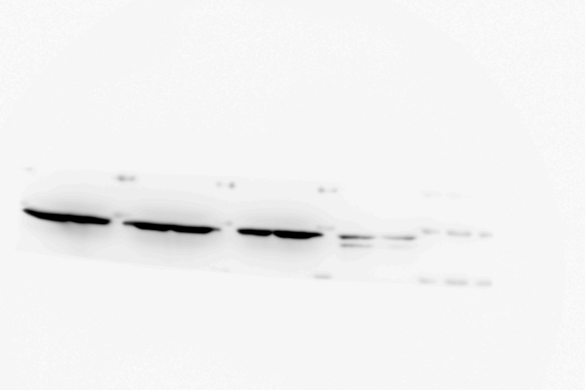

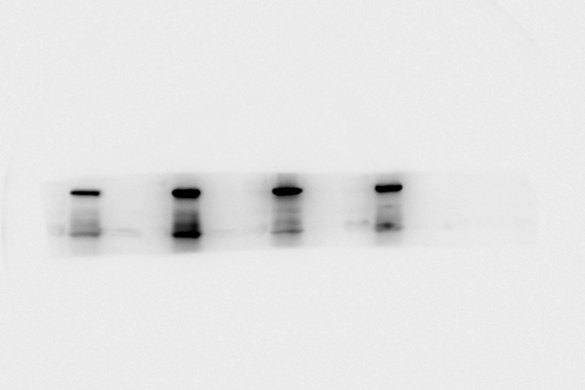


JNK pJNK


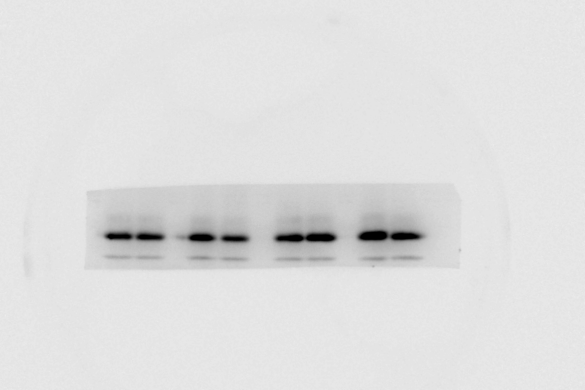

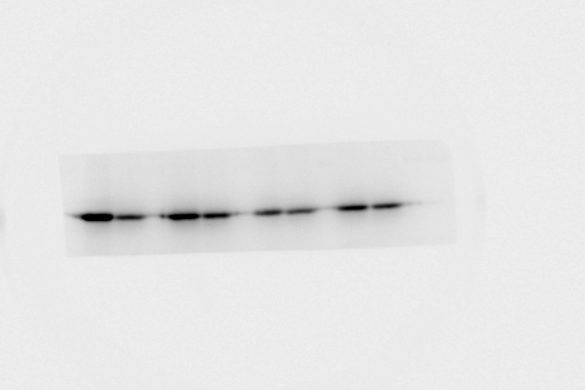


**Fig. S7F**

β-actin HA (Atx2)


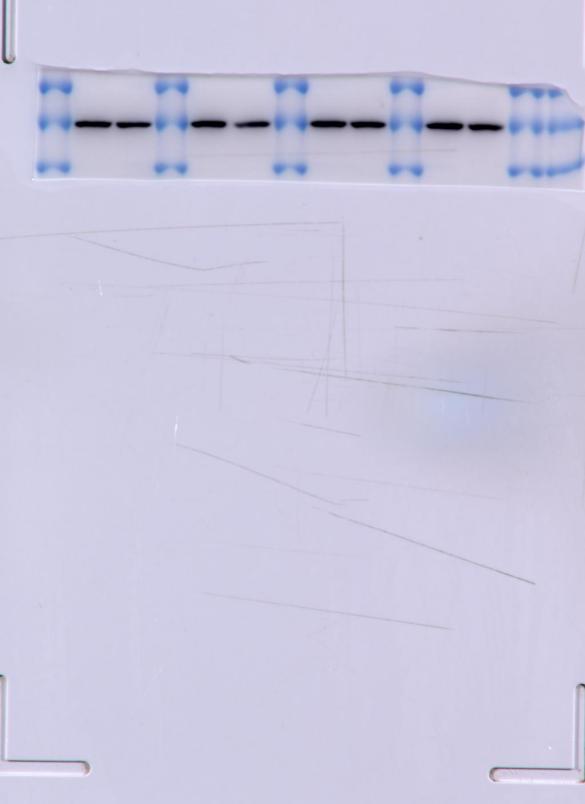

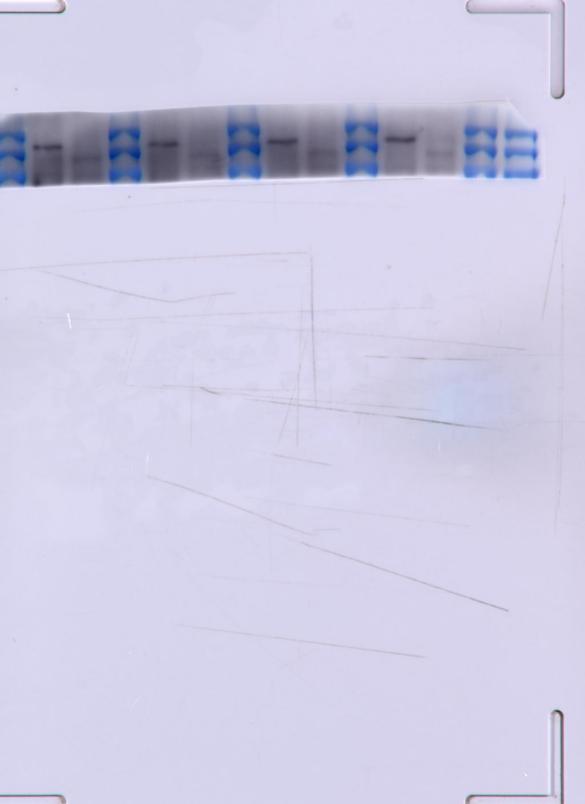


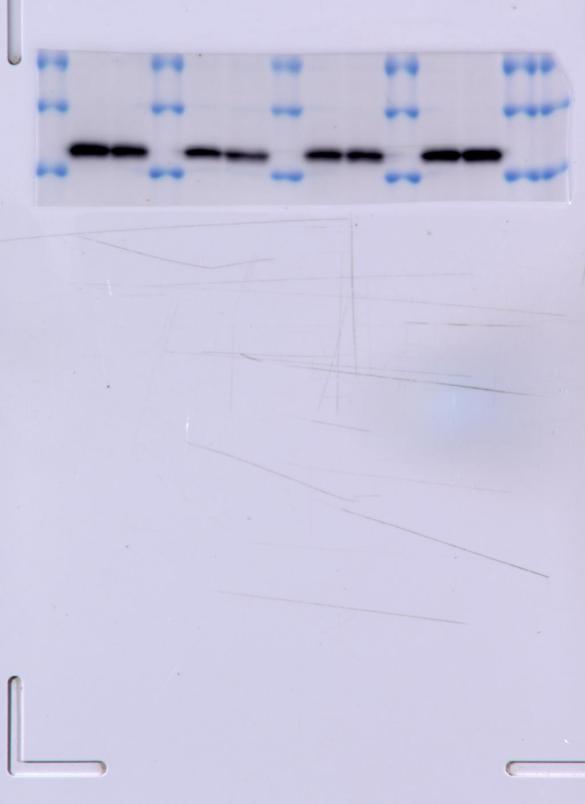
GFP

**Fig. S9C**

β-actin JNK


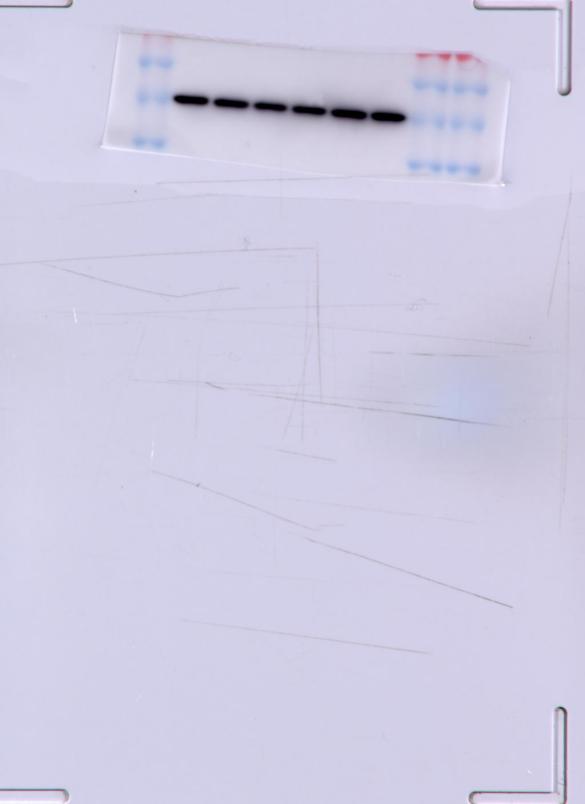

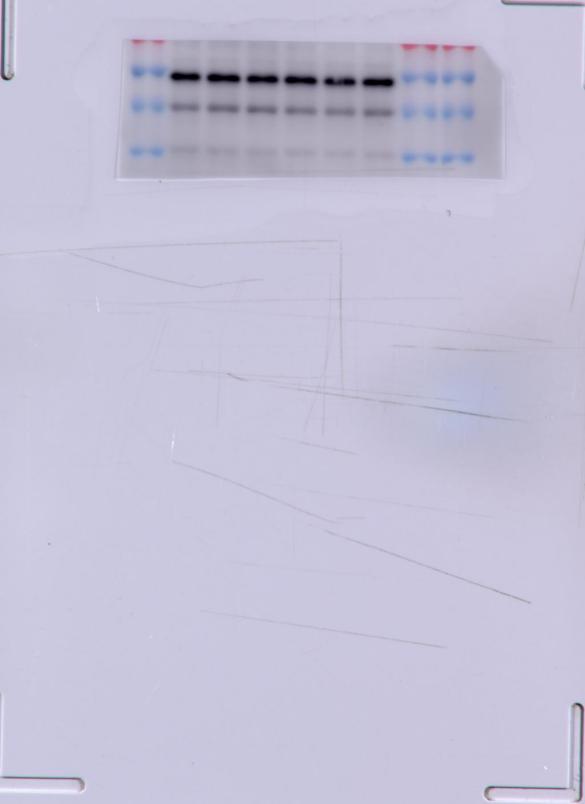


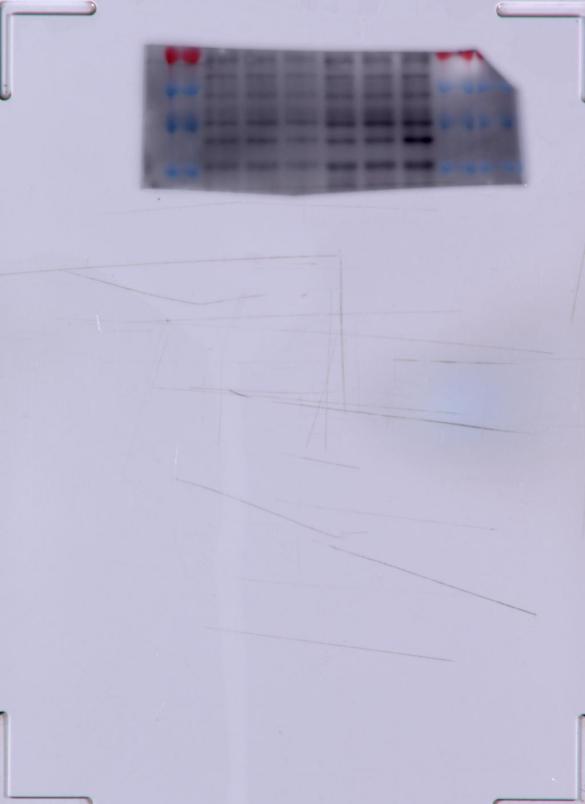
pJNK
